# Salinization and arsenic contamination of surface water in southwest Bangladesh

**DOI:** 10.1186/s12932-017-0042-3

**Published:** 2017-09-11

**Authors:** John C. Ayers, Gregory George, David Fry, Laura Benneyworth, Carol Wilson, Leslie Auerbach, Kushal Roy, Md. Rezaul Karim, Farjana Akter, Steven Goodbred

**Affiliations:** 1 Department of Earth & Environmental Sciences, Vanderbilt University, Nashville, TN 37240 USA; 2 Department of Geology and Geophysics, Louisiana State University, Baton Rouge, LA 70803 USA; 3 Environmental Science Discipline, Khulna University, Khulna, 9208 Bangladesh; 4 Department of Earth & Environmental Sciences, Vanderbilt University, PMB 351805, 2301 Vanderbilt Place, Nashville, TN 37235-1805 USA

**Keywords:** Salinization, Arsenic, Aquaculture, Water chemistry, Bangladesh

## Abstract

To identify the causes of salinization and arsenic contamination of surface water on an embanked island (i.e., polder) in the tidal delta plain of SW Bangladesh we collected and analyzed water samples in the dry (May) and wet (October) seasons in 2012–2013. Samples were collected from rice paddies (wet season), saltwater ponds used for brine shrimp aquaculture (dry season), freshwater ponds and tidal channels (both wet and dry season), and rainwater collectors. Continuous measurements of salinity from March 2012 to February 2013 show that tidal channel water increases from ~0.15 ppt in the wet season up to ~20 ppt in the dry season. On the polder, surface water exceeds the World Health Organization drinking water guideline of 10 μg As/L in 78% of shrimp ponds and 27% of rice paddies, raising concerns that produced shrimp and rice could have unsafe levels of As. Drinking water sources also often have unsafe As levels, with 83% of tubewell and 43% of freshwater pond samples having >10 μg As/L. Water compositions and field observations are consistent with shrimp pond water being sourced from tidal channels during the dry season, rather than the locally saline groundwater from tubewells. Irrigation water for rice paddies is also obtained from the tidal channels, but during the wet season when surface waters are fresh. Salts become concentrated in irrigation water through evaporation, with average salinity increasing from 0.43 ppt in the tidal channel source to 0.91 ppt in the rice paddies. Our observations suggest that the practice of seasonally alternating rice and shrimp farming in a field has a negligible effect on rice paddy water salinity. Also, shrimp ponds do not significantly affect the salinity of adjacent surface water bodies or subjacent groundwater because impermeable shallow surface deposits of silt and clay mostly isolate surface water bodies from each other and from the shallow groundwater aquifer. Bivariate plots of conservative element concentrations show that all surface water types lie on mixing lines between dry season tidal channel water and rainwater, i.e., all are related by varying degrees of salinization. High As concentrations in dry season tidal channel water and shrimp ponds likely result from groundwater exfiltration and upstream irrigation in the dry season. Arsenic is transferred from tidal channels to rice paddies through irrigation. Including groundwater samples from the same area (Ayers et al. in Geochem Trans 17:1–22, 2016), principal components analysis and correlation analysis reveal that salinization explains most variation in surface water compositions, whereas progressive reduction of buried surface water by dissolved organic carbon is responsible for the nonconservative behavior of S, Fe, and As and changes in Eh and alkalinity of groundwater.

**Electronic supplementary material:**

The online version of this article (doi:10.1186/s12932-017-0042-3) contains supplementary material, which is available to authorized users.

## Introduction

The Ganges–Brahmaputra–Meghna delta in Bangladesh is the world’s largest and most densely populated river delta, supporting approximately 160 million people. Water quality in SW Bangladesh is threatened by contamination of water by arsenic, dissolved salts, and pathogens, especially during the long dry season, which lasts from November to May [1]. In the 1990s it was discovered that groundwater from 6 to 10 million tubewells in Bangladesh had As concentrations higher than the World Health Organization (WHO) guideline for drinking water of 10 μg/L [2], meaning that more than 57 million people were exposed to unsafe levels of As [3]. Arsenic is a carcinogen to humans and exposure from drinking contaminated water can increase the risk of skin, lung, bladder and kidney cancers, hypertension, diabetes, peripheral vascular disease, and skin lesions [4]. Arsenic present in soil and irrigation (rice paddy) water can also be incorporated into rice, presenting another exposure risk [5–7].

Salinization of surface water and groundwater is another problem in the coastal area of southwest Bangladesh, which similarly leads to negative health effects and reduced agricultural production [8]. Long term exposure to saline drinking water can cause hypertension [9]. In southwest Bangladesh high drinking water salinity has also been linked to relatively high rates of preeclampsia and gestational hypertension, with the latter occurring at higher rates in the dry season than in the wet season [10]. High salinity in irrigation water and soil also decreases crop yields [11]. For example, when irrigation water exceeds 5 ppt salinity, crop yields decrease as much as 50% [8].

Ayers et al. [12] examined the causes of salinization and arsenic contamination of groundwater resources in Polder 32 in southwest Bangladesh and found that salts in the shallow aquifer groundwater were derived from connate water, whereas sedimentary As was mobilized by reductive dissolution of ferric oxyhydroxides. A related modeling paper also demonstrated that variance in local groundwater salinity could be explained simply by the dilution of connate groundwater having an initial mean-annual salinity through the slow, localized recharge of fresh surface water [13]. In a paper on water security for Polder 32, Benneyworth et al. [1] found that local drinking water sources, including groundwater, rainwater, and surface ponds, commonly exceed Bangladesh government guidelines of 2 mS/cm for specific conductivity (a proxy for salinity) and 50 μg/L As, which raises health concerns. The complementary paper presented here further analyzes the chemical composition of the various surface water sources in Polder 32 (including freshwater pond, rice paddy, shrimp pond, tidal channel, and rainwater), and examines the compositional relationships between groundwater and surface waters and the processes that affect their compositions, with a focus on identifying the sources of dissolved salts and arsenic.

### Geographic setting

**Fig. 1:**
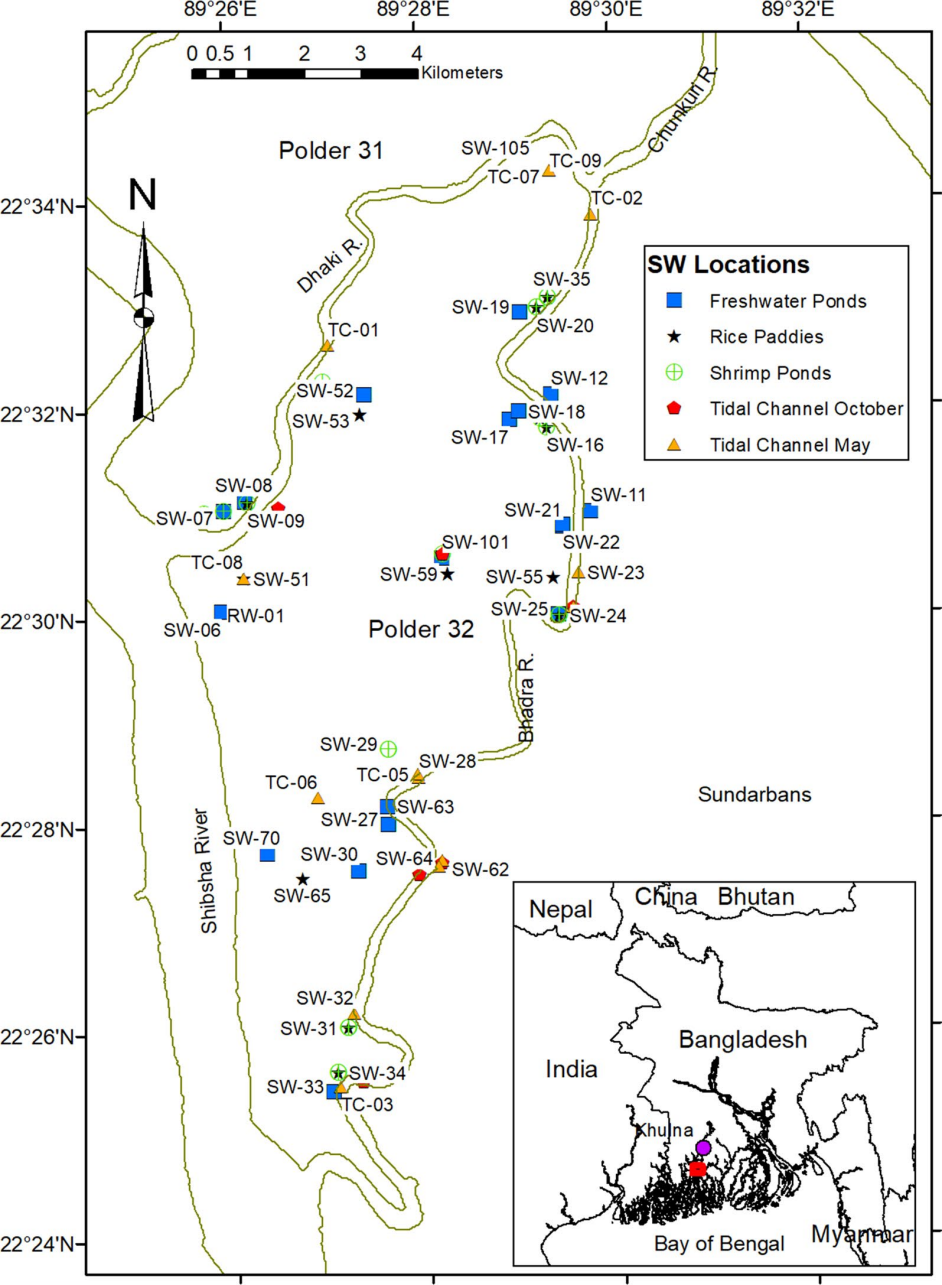
Locations of surface water sampling sites classified by water type. In the *inset map* the *red square* marks the location of the larger map

The study area is located within the ‘abandoned’ tidal delta plain of southwest Bangladesh (Fig. 1), which covers ~20,000 km^2^ in a dense network of tidal channels and intertidal islands previously colonized by mangrove vegetation. This region was initially formed as part of the active Ganges rivermouth in the mid-Holocene, before that river migrated eastward in the late Holocene [14, 15]. Since that time, waning fluvial discharge from the main distributaries [16] has caused the subaerial landscape to be maintained by onshore tidal sediment transport [17, 18].

After major floods in 1954 and 1955 followed by years of famine, many of the tidal islands in this area were converted to polders in the 1960s and 1970s by building high earthen embankments around their perimeters for flood control and to increase arable land for rice cultivation [19]. This eliminated regular tidal inundation of the landscape, thereby depriving embanked islands of the sediment normally supplied by these flood waters. Over time subsidence, tidal amplification and channel aggradation have increased the elevation of waters within tidal channels relative to the polders [20, 21]. While polder elevation remains above mean sea level, tidal channels located outside the embankments are aggrading to approximately mean high water, making it difficult to keep conduits and connecting channels deep enough to drain the polder [20, 22]. When embankment failures occur, either through storm surges or channel migration, the landscape is exposed to exacerbated tidal flooding and waterlogging, potentially causing salinization of surface water and groundwater. Exchange of water between tidal channels and polders is facilitated by, or results from, average changes in tidal channel surface elevation of 3–4 m between spring low and high tide near Polder 32 [20].

The focus of this study is Polder 32 in Khulna district, Dacope Upazila, about 30 km south of the city of Khulna and 60 km north of the Bay of Bengal (Fig. 1). The polder is 19.3 by 7.1 km with a total area of 68.2 km^2^ and has a population of roughly 40,000. It is surrounded by tidal channels, across which lie the Sundarbans mangrove forest in the south, Polder 33 to the east, and Polder 31 to the north and west. Surface sediments and soils in Polder 32 are silt-dominated and clay-rich, forming an impermeable mud cap [12, 13].

### Potential causes of surface water salinization

Areas of southwest Bangladesh have experienced chronic problems with surface water salinization. One potential cause of salinization of freshwater ponds and rice paddy water is the dry-season diversion of the Ganges River by the Farraka Barrage that was completed in India in 1975 [16]. This diversion causes a large decrease in dry season discharge and increase in salinity in the Ganges River downstream of the Farraka Barrage and in the Gorai River that branches off the Ganges and was historically the principal dry-season source of freshwater to southwest Bangladesh [23]. With decreased freshwater discharge, the surface water salinity front migrates further inland during the dry season than previously. This study evaluates the impacts of two other potentially important causes of surface water salinization: tidal channel water inundation following embankment breaches, and brine shrimp aquaculture, which requires flooding the landscape with saline ground- or surface-waters.

#### Tidal channel water inundation

**Fig. 2:**
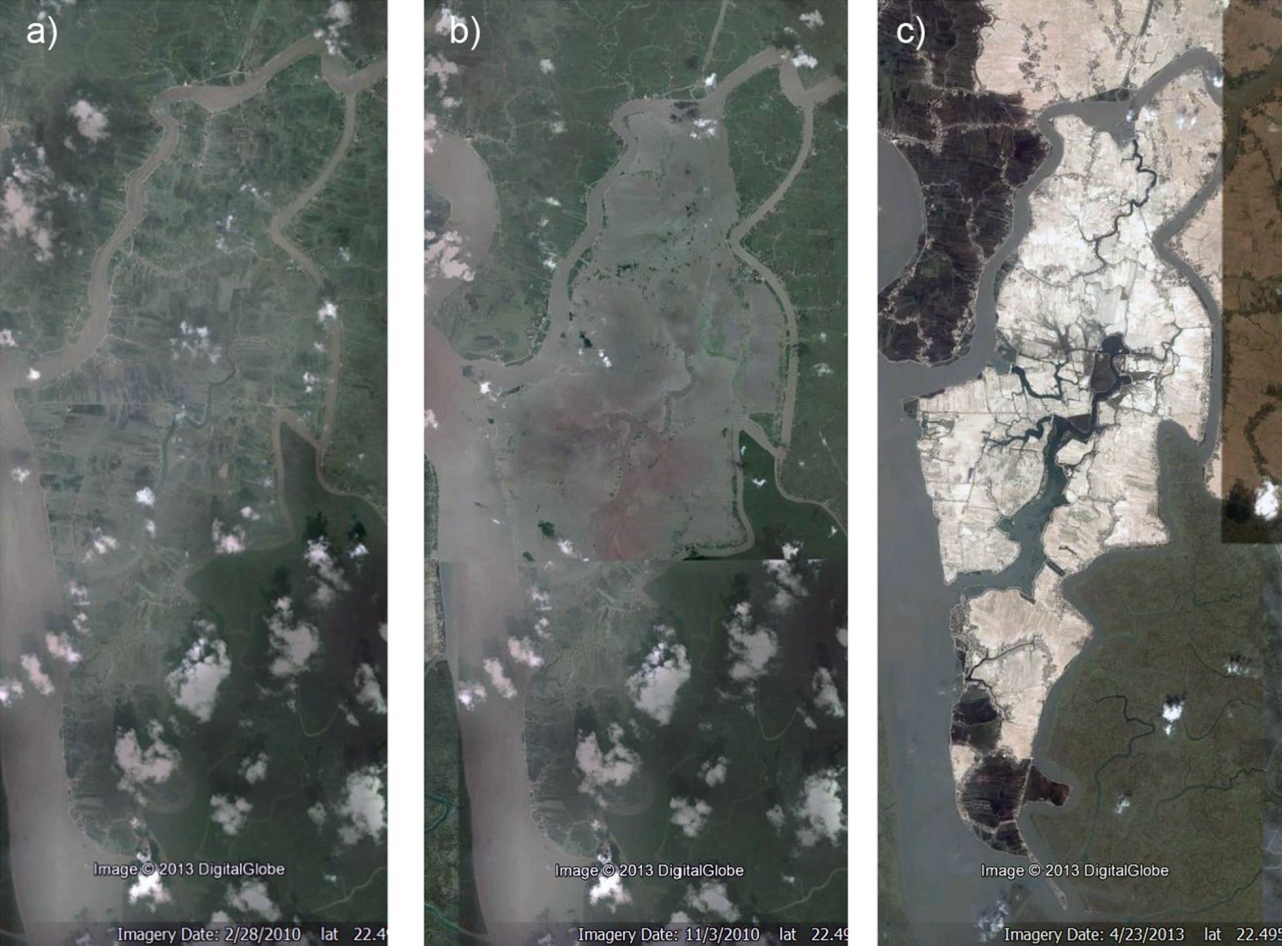
Google Earth satellite photos of Polder 32. **a** Wet season, October 13, 2008. **b** Polder partially inundated after Cyclone Aila, November 3, 2010. **c** Dry season, April 6, 2013

Polders in southwest Bangladesh are highly susceptible to storm surges during cyclones [24]. Roughly ¾ of Polder 32 (~51.2 km^2^) was inundated for 2 years following the failure of five embankments during Cyclone Aila in May 2009 (Fig. 2b). During that time, the polder was submerged to a mean depth of 1 m for an average of ~10 h/day [20]. Roughly 40 cm of silty sediments were deposited on Polder 32 during the 2 years of inundation [20]. Surface water ponds were inundated and contaminated by salts and pathogens, resulting in a severe shortage of safe drinking water [24].

Long-term inundation with brackish water from the surrounding tidal channels and deposition of salt-rich sediments may lead to salinization of surface water bodies in polders. Tidal channel waters in this region are generally saline during the dry season and relatively fresh during the wet season [25]. Sediments deposited during the dry season likely contained saline pore water. On Polder 32, once inundation ceased following embankment repair in approximately May 2011, it presumably would take time for salts to be flushed out of the low permeability sediments; during that time surface water bodies would be expected to have higher than normal salinities due to leaching. This process has not been documented previously. To rectify this, beginning in May 2012 we measured the salinity of water from freshwater ponds and rice paddies on Polder 32 in areas that experienced long-term inundation with brackish tidal channel water and in control areas that were not inundated.

#### Brine shrimp aquaculture

**Fig. 3:**
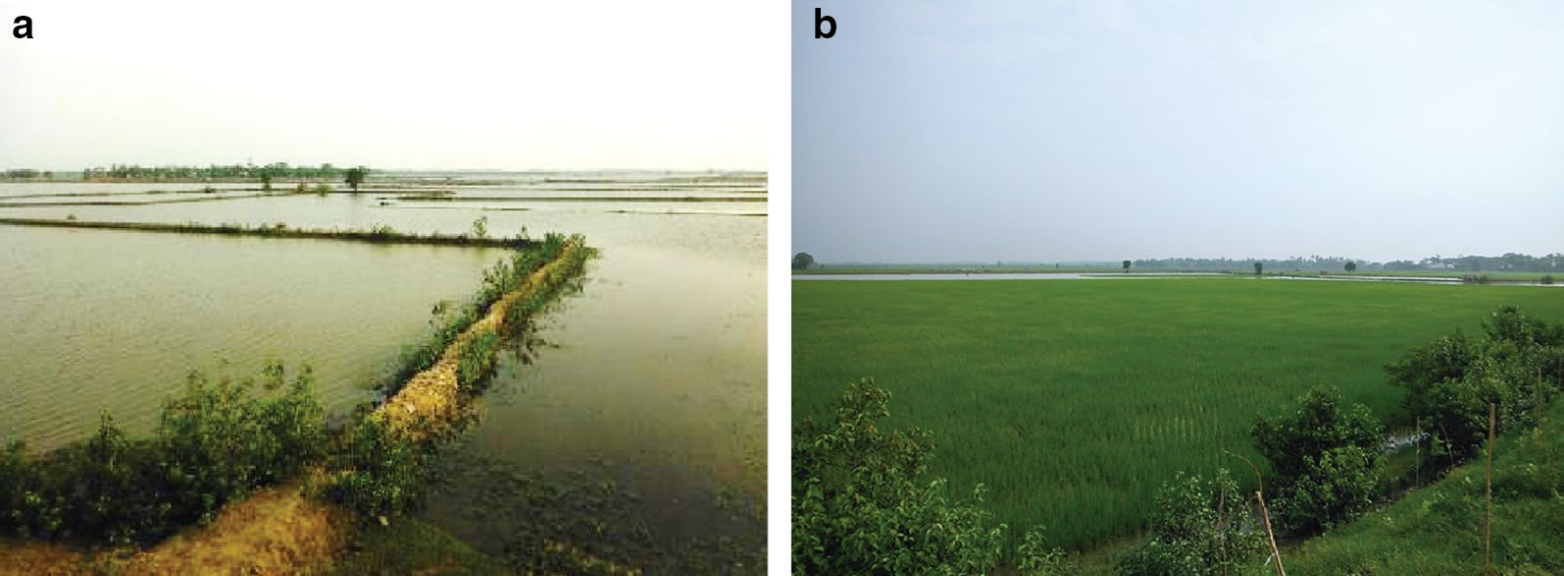
Photos of the same area of Polder 32, when it was a **a** Shrimp pond, May 2012, versus **b** rice paddy, October 2012

During the summer wet season (June to November) rice is grown in paddies in southwest Bangladesh. However, high salinity of surface water and groundwater precludes production of rice in the dry season (December to May). Beginning in 1985, brine shrimp aquaculture was introduced to the region as a profitable use for fallow lands during the dry season [11]. It is now common practice in southwest Bangladesh to rotate land use between shrimp farming in the dry season and salt-tolerant rice farming in the wet season (Fig. 3; [26]). At the time of this study, brine shrimp ponds on Polder 32 were constructed into surface soils and generally located adjacent to tidal channels to facilitate the exchange of saline water through sluice gates (Fig. 3).

Dry season shrimp farming in southwest Bangladesh has been found to cause salinization of shrimp pond water and soil [27]. Over a 15 year period farms that practiced dry season shrimp farming in southwest Bangladesh showed increases in soil salinity and decreased wet season rice yields [11]. Shrimp farms can also cause salinization of adjacent farmlands if they improperly discharge saltwater during seasonal change-out of brine shrimp aquaculture ponds [26]. However, no studies in this region have evaluated the impacts of dry season brine shrimp aquaculture on water salinity in nearby freshwater ponds or in rice paddies during the wet season.

This study tests the hypotheses that shrimp farming and tidal channel water inundation cause salinization of water in rice paddies and freshwater ponds near Polder 32. In addition, it presents measurements of As concentrations in various surface water types and explores geochemical relationships between surface waters and previously reported groundwater compositions [12].

### Potential causes of surface water arsenic contamination

Although As contamination of groundwater in Bangladesh has been thoroughly studied, less is known about the extent and cause(s) of As contamination of surface water [28]. Generally, arsenic concentrations are expected to be low in oxidized surface waters and can be high in reduced groundwaters [29] because arsenic sorbs to ferric oxydroxides in sediments under oxidizing conditions. One potential source of arsenic is leaching from rocks during chemical weathering. Pyrite in coals seams in the Himalaya is believed to be an important source of As [30, 31]. Released ferrous iron is oxidized to form ferric oxyhydroxides that sorb the As and allow it to be transported by rivers, which deposit As-rich sediments in floodplains. Arsenic could be leached from these sediments into water in freshwater ponds, shrimp ponds, or rice paddies. Arsenic in rivers could also be leached from riverbank sediments that have high arsenic concentrations due to discharge of reducing, As-rich groundwater during the dry season [32, 33]. Finally, arsenic could derive from groundwater used for irrigation upstream.

## Methods

Samples were collected at the peaks of the dry season (May) and wet season (October) in years 2012 and 2013 throughout the study area shown in Fig. 1. Most sample locations were chosen close to populated areas, principally around the perimeter of Polder 32 where residents had constructed surface ponds and tubewells. Some water samples were also collected on adjacent Polders 31 and 33. Sample locations were measured with a horizontal accuracy of 50 cm using a Trimble GeoXT 6000 (Table 1). Data was stored and analyzed in ESRI ArcGIS 10.4.

**Table 1:** Sample locations

Location	Longitude (°)^a^	Latitude (°)	Inundated	Location	Longitude (°)	Latitude (°)	Inundated
RW-04	89.438681	22.462538		SW-32	89.453665	22.436448	
SW-06	89.431902	22.501151	Y	SW-33	89.449971	22.423910	N
SW-07	89.432446	22.517320	N	SW-34	89.450806	22.427044	Y
SW-08	89.436222	22.518878	N	SW-35	89.489165	22.551083	N
SW-09	89.436607	22.518719	N	SW-36	89.469382	22.460860	
SW-10	89.429141	22.516978		SW-50	89.441961	22.517735	
SW-100	89.449913	22.537869		SW-51	89.435810	22.506626	Y
SW-101	89.470047	22.509296		SW-52	89.457070	22.535705	Y
SW-103	89.470224	22.509981		SW-53	89.456294	22.532590	Y
SW-105	89.483568	22.573514		SW-55	89.489258	22.506109	Y
SW-11	89.495879	22.516575	N	SW-56	89.492793	22.501237	
SW-12	89.489366	22.535426	N	SW-58	89.470224	22.509981	
SW-13	89.488781	22.532755		SW-59	89.471037	22.506890	Y
SW-14	89.487495	22.531589	Y	SW-60	89.471037	22.506890	Y
SW-16	89.488550	22.530110	Y	SW-61	89.455115	22.425500	
SW-17	89.482202	22.531417	Y	SW-62	89.469357	22.460383	
SW-18	89.483725	22.532724	Y	SW-63	89.459957	22.469587	Y
SW-19	89.484203	22.548704	Y	SW-64	89.465396	22.458601	
SW-20	89.487210	22.549411	Y	SW-65	89.445202	22.458278	Y
SW-21	89.491094	22.514213	Y	SW-70	89.439094	22.462249	
SW-22	89.491073	22.514405	Y	TC-01	89.450950	22.543850	
SW-23	89.493640	22.506752		TC-02	89.496848	22.564229	
SW-24	89.490216	22.499947	N	TC-03	89.451185	22.424650	
SW-25	89.490032	22.500100	Y	TC-04	89.468683	22.459838	
SW-27	89.460058	22.466748	Y	TC-05	89.465426	22.474728	
SW-28	89.465479	22.474240		TC-06	89.448070	22.471190	
SW-29	89.460353	22.478736		TC-07	89.489775	22.571341	
SW-30	89.454787	22.459361	N	TC-08	89.435810	22.506626	
SW-31	89.452650	22.434218	Y	TC-09	89.481467	22.531006	

^a^Datum for location is WGS 1984. Freshwater pond and rice paddy sites inundated following Cyclone Aila are indicated with “Y”

### Field measurements and sample collection

All field and laboratory methods are described in Ayers et al. [12]. Briefly, in 2012 and May 2013 a Hach Hydrolab 4a was used in the field to measure pH, oxidation–reduction potential Eh in millivolts (mV), temperature in degrees Celsius (°C), and specific conductivity (SpC) in millisiemens per centimeter (mS/cm). In October 2013 a Hach Hydrolab DS5 was used to make the same measurements.

Platinum electrodes like those in the Hydrolab units typically only respond to a few electroactive species present at concentrations greater than ~10^−5^ molal in natural waters, usually only Fe^2+^/Fe^3+^ [34]. Thus, Eh measurements are most useful for distinguishing oxic versus anoxic conditions. Comparison of Eh measurements for surface water samples in this study (Table 2) with those of groundwater samples measured using the same equipment during the same time periods [12] shows that Eh measurements made with the Hydrolab can distinguish between oxic surface waters and anoxic groundwaters.

**Table 2:** Water sample compositions

Loc	Date	Type	T (°C)	Eh (mV)	pH	SpC (mS/cm)	Salinity (ppt)	Al	As	B	Ba	Ca	Fe	K	Li	Mg
Blank 1	10/19/2012	Blank								0.002	0.002	0.2		0.0		0.1
Blank 2	10/18/2012	Blank									0.002	0.007		0.004		
Blank 3	5/13/2012	Blank							0.006		0.002	0.1		0.1		0.1
Blank 4	5/13/2012	Blank						0.1		0.053	0.008	1.8	0.014	0.4		0.8
SW-06	5/14/2012	FP	34.6	436	8.06	1.84	0.77	0.06	0.007	0.09	0.07	56	0.116	18.1	0.007	39.8
SW-06	10/15/2012	FP	31.8	360	8.66	1.27	0.55	0.02	0.006	0.10	0.04	46		11.1	0.003	25.2
SW-06	10/24/2013	FP	28.3	170	7.7	1.09	0.50	0.02	0.006	0.09	0.02	33		9.5		21.5
SW-07	10/15/2012	FP	29.6	363	8.64	*5.14*	2.51		0.006	0.45	0.04	50		42.9	0.013	92.9
SW-08	5/16/2012	FP	31.1	395	7.69	1.76	0.79	0.01	0.003	0.10	0.05	73	0.007	20.7	0.011	40.7
SW-08	10/15/2012	FP	30.2	348	8.6	1.41	0.63	0.02	0.006	0.13	0.03	67		13.2	0.004	32.5
SW-101	5/7/2013	FP	34.1	167	8.56	*4.48*	1.98	0.08	*0.059*	0.86	0.15	248	0.025	95.9	0.024	250.7
SW-11	5/17/2012	FP	32.7	397	7.39	1.29	0.55	0.04	*0.013*	0.05	0.10	59	0.025	23.5	0.009	34.1
SW-12	5/17/2012	FP	35.9	396	8.45	1.02	0.41	0.01	*0.020*	0.02	0.03	70	0.018	27.4	0.008	32.8
SW-12	10/16/2012	FP	31.4	372	8.83	1.79	0.79	0.04	0.004	0.10	0.04	105	0.003	26.0	0.005	50.2
SW-17	5/18/2012	FP	33.5	365	7.37	*2.23*	0.96	0.01	0.009	0.15	0.10	45	0.018	27.6	0.008	43.4
SW-17	10/16/2012	FP	32.9	305	8.61	*2.23*	0.97		*0.037*	0.22	0.04	54	0.003	16.2	0.007	46.8
SW-17	5/7/2013	FP	32.2	93	9.16	*2.61*	1.16	0.03	0.006	0.27	0.08	45	0.015	24.5		49.5
SW-17	10/25/2013	FP	29.3	126	7.4	1.90	0.88	0.01	*0.011*	0.18	0.01	32		15.4		36.1
SW-18	5/18/2012	FP	34.3	350	7.1	*2.29*	0.97	0.00	*0.019*	0.11	0.08	60	0.019	20.1	0.007	42.5
SW-18	10/16/2012	FP	32.6	261	8.95	1.74	0.75	0.03	0.009	0.11	0.05	63	0.009	11.9	0.003	33.4
SW-18	5/7/2013	FP	32.9	23	8.74	*2.19*	0.95	0.04	*0.021*	0.19	0.08	67	0.010	16.4		40.4
SW-18	10/25/2013	FP	29.3	184	7.75	1.28	0.59	0.02	0.009	0.10	0.03	44		9.0		22.7
SW-19	5/19/2012	FP	31.8	411	8.14	1.88	0.83	0.01	*0.049*	0.06	0.09	88	0.007	30.3	0.011	35.9
SW-19	10/16/2012	FP	29.8	378	8.62	1.54	0.70	0.02	*0.027*	0.09	0.05	73		19.8	0.004	28.6
SW-19	5/6/2013	FP	30.7	124	9.28	*2.36*	1.07	0.04	*0.027*	0.15	0.14	113	0.006	31.8	0.013	41.8
SW-19	10/23/2013	FP	29.6	170	8.18	1.96	0.91	0.04	*0.020*	0.10	0.06	86	0.012	22.2		36.6
SW-21	5/20/2012	FP	33.1	414	7.45	1.65	0.71	0.01	0.009	0.07	0.06	51	0.002	22.2	0.006	36.9
SW-21	10/17/2012	FP	29.9	379	8.07	1.23	0.55	0.02	0.008	0.11	0.04	42	0.002	14.1	0.004	28.1
SW-22	5/20/2012	FP	33.0	415	7.55	1.65	0.71	0.01	*0.015*	0.07	0.05	49	0.003	21.8	0.008	36.9
SW-22	10/26/2013	FP	26.8	213	7.36	1.14	0.54	0.02	0.003	0.09	0.03	36		11.9		25.5
SW-25	5/20/2012	FP	38.0	415	7.88	*4.35*	1.79	0.02	*0.012*	0.21	0.08	91		39.1	0.012	88.8
SW-25	10/17/2012	FP	30.1	286	8.97	1.87	0.85	0.01	0.006	0.09	0.02	42	0.005	10.8	0.003	30.2
SW-25	5/7/2013	FP	33.5	125	8.78	*3.31*	1.46	0.02	*0.038*	0.23	0.01	50	0.007	24.4		66.3
SW-27	5/21/2012	FP	33.5	433	7.28	*2.92*	1.28	0.00	0.006	0.17	0.06	42	0.002	29.9	0.008	52.9
SW-27	10/19/2012	FP	30.4	266	8.2	1.88	0.85	0.01	*0.011*	0.17	0.03	30	0.004	15.9	0.003	32.6
SW-27	5/5/2013	FP	30.0	86	7.95	*2.56*	1.19	0.02		0.22	0.06	43		21.0		36.5
SW-27	10/26/2013	FP	26.8	140	7.3	1.21	0.58	0.01	0.004	0.11	0.01	18	0.008	10.2		20.7
SW-30	5/22/2012	FP	31.8	410	8.73	2.00	0.89	0.00	*0.017*	0.09	0.07	39	0.005	21.5	0.007	32.3
SW-30	10/19/2012	FP	32.3	336	8.64	1.47	0.63	0.02	0.007	0.14	0.06	45	0.002	13.9	0.004	29.5
SW-30	5/8/2013	FP	30.6	58	8.36	*2.17*	0.99	0.03	0.006	0.21	0.09	64	0.008	20.7		40.3
SW-33	5/23/2012	FP	31.0	444	7.94	*8.14*	3.98	0.02	*0.038*	0.58	0.11	120	0.011	92.3	0.012	167.7
SW-33	10/18/2012	FP	32.8	275	9.21	*2.48*	1.09	0.01	0.008	0.28	0.05	59	0.013	28.2	0.007	49.0
SW-33	5/5/2013	FP	31.6	163	8.48	*6.29*	2.99	0.04		0.67	0.13	106	0.010	70.7	0.008	127.0
SW-33	10/27/2013	FP	28.4	96	8.05	1.87	0.88	0.02	0.003	0.18	0.03	41		19.7		33.1
SW-52	10/15/2012	FP	32.0	349	8.97	1.41	0.61	0.02	*0.012*	0.12	0.05	58		14.4	0.005	32.1
SW-52	10/24/2013	FP	31.0	152	8.44	1.30	0.57	0.03	0.007	0.10	0.04	58	0.002	13.1		29.3
SW-63	10/19/2012	FP	29.1	218	8.53	1.89	0.88	0.05	0.007	0.16	0.07	35	0.003	23.5	0.003	28.2
SW-70	10/27/2013	FP	28.4	143	7.72	1.02	0.47	0.02	0.003	0.09	0.02	37		10.0		25.6
SW-09	10/15/2012	RP	28.6	356	8.17	*3.91*	1.92		*0.015*	0.51	0.04	50		33.3	0.012	68.1
SW-14	10/25/2013	RP	28.7	154	7.41	*2.88*	1.38					1		0.2		0.6
SW-16	10/16/2012	RP	37.8	322	9.45	0.67	0.26	0.06	0.005	0.08	0.02	31	0.074	9.5	0.004	16.5
SW-20	10/16/2012	RP	29.2	391	8.01	1.73	0.80	0.02	0.007	0.17	0.05	57	0.003	16.7	0.007	39.2
SW-20	10/23/2013	RP	28.9	188	7.32	0.93	0.42	0.02		0.09	0.03	44		7.0		21.5
SW-24	10/17/2012	RP	30.4	295	8.61	1.45	0.65	0.02	0.003	0.14	0.04	36	0.005	15.3	0.004	28.5
SW-31	10/18/2012	RP	30.7	244	8.24	2.00	0.90	0.02	*0.011*	0.19	0.04	42	0.002	19.7	0.007	41.4
SW-31	10/27/2013	RP	25.3	173	7.25	1.61	0.81	0.01		0.16	0.02	24		15.7		27.8
SW-34	10/18/2012	RP	33.4	261	9.25	*2.43*	1.05	0.04	*0.039*	0.24	0.06	49	0.011	18.6	0.008	48.4
SW-35	10/16/2012	RP	28.5	393	8.31	0.82	0.38	0.01	0.002	0.13	0.04	31	0.003	14.5	0.004	25.6
SW-35	10/23/2013	RP	28.7	190	7.27	1.51	0.70	0.02	0.002	0.15	0.05	53	0.008	10.3		29.8
SW-51	10/15/2012	RP	33.6	309	8.72	1.57	0.66	0.03	0.003	0.15	0.04	51	0.009	13.3	0.004	37.6
SW-53	10/15/2012	RP	30.5	349	8.19	0.66	0.29	0.01	0.002	0.06	0.04	34		7.0	0.003	16.6
SW-53	10/25/2013	RP	29.8	189	7.36	1.51	0.69	0.02	0.002	0.13	0.04	48		8.3		31.0
SW-55	10/17/2012	RP	29.3	228	8.06	1.97	0.92	0.02	*0.011*	0.20	0.05	56	0.004	17.0	0.005	45.4
SW-59	10/17/2012	RP	32.5	333	8.89	*4.33*	1.97	0.02	0.005	0.30	0.07	107	0.006	31.3	0.008	91.7
SW-60	10/17/2012	RP	32.5	333	8.89	*4.33*	1.97	0.01	0.003	0.30	0.08	106	0.014	30.9	0.008	89.8
SW-65	10/19/2012	RP	32.5	259	8.58	1.34	0.58	0.02	0.003	0.11	0.04	43	0.009	10.9	0.004	29.5
RW-02	10/26/2013	RW	22.5	212	6.56	0.01	0.01	0.01			0.02	1	0.011	0.1		0.1
RW-03	10/26/2013	RW	22.3	213	6.6	0.01	0.01					1		0.1		0.1
RW-04	10/27/2013	RW	26.3	95	8.72	0.08	0.04	0.12			0.00	10		3.2		0.3
SW-07	5/16/2012	SP	30.2	419	8.35	*31.34*	17.46	0.05	0.002	2.23	0.24	299	0.006	368.6	0.007	771.3
SW-09	5/16/2012	SP	30.6	434	8.23	*28.03*	15.32	0.05	*0.012*	2.08	0.30	230	0.005	324.8	0.006	662.3
SW-10	5/16/2012	SP	32.2	445	7.68	*25.32*	13.27	0.05	*0.015*	1.78	0.26	240	0.017	296.1	0.007	607.0
SW-100	5/4/2013	SP	31.8	77	7.95	*28.16*	15.03	0.07	*0.053*	2.86	0.35	252	0.007	282.3	0.024	668.9
SW-103	5/7/2013	SP	34.1	168	8.79	*8.13*	3.75	0.08	*0.023*	0.73	0.16	274	0.031	78.3	0.025	201.7
SW-14	5/18/2012	SP	31.7	430	8.58	*21.17*	11.03	0.07	*0.033*	0.90	0.46	408	0.015	166.3	0.015	478.3
SW-16	5/18/2012	SP	33.4	449	8.03	*32.00*	16.76	0.05	*0.010*	2.49	0.28	259	0.007	385.1	0.007	765.1
SW-20	5/19/2012	SP	34.0	459	7.94	*30.57*	15.76	0.05	0.004	2.22	0.45	261	0.020	347.9	0.009	724.0
SW-20	5/6/2013	SP	30.1	174	8.5	*27.97*	15.44	0.07	*0.043*	2.48	0.23	246		256.8	0.025	614.5
SW-24	5/20/2012	SP	39.2	451	8.02	*27.76*	12.87	0.05	*0.021*	1.91	0.40	235	0.007	308.2	0.007	633.8
SW-24	5/7/2013	SP	33.3	174	7.54	*28.35*	14.70	0.06	*0.024*	2.50	0.32	245	0.009	279.6	0.024	684.1
SW-29	5/21/2012	SP	34.8	473	8.33	*22.25*	10.96	0.05	0.010	1.26	0.27	391	0.005	225.9	0.011	520.6
SW-29	5/6/2013	SP	30.3	67	8.71	*21.65*	11.62	0.06		1.01	0.80	250	0.010	96.4	0.024	455.4
SW-31	5/22/2012	SP	35.9	426	8.11	*35.74*	18.05	0.06	*0.014*	2.76	0.21	287	0.007	432.7	0.006	847.1
SW-31	5/5/2013	SP	26.3	95	7.57	*34.82*	21.28	0.08	*0.052*	3.32	0.19	303	0.031	321.3	0.030	811.4
SW-34	5/23/2012	SP	31.4	474	7.33	*35.32*	19.44	0.05	*0.025*	2.70	0.22	287	0.009	416.3	0.008	829.1
SW-35	5/19/2012	SP	31.4	468	7.6	*30.89*	16.77	0.06	*0.016*	2.22	0.35	297	0.027	350.4	0.007	721.3
SW-35	5/6/2013	SP	30.5	171	8.41	*29.36*	16.15	0.07	*0.040*	2.98	0.32	237	0.019	270.4	0.026	674.0
SW-105	10/22/2013	TC	28.4	180	7.29	0.68	0.31	0.02	0.003	0.05	0.03	40		4.5		15.8
SW-50	10/15/2012	TC	30.5	348	8.03	0.52	0.22	0.01	0.002	0.05	0.04	31		5.5	0.002	11.2
SW-58	10/17/2012	TC	33.2	282	8.89	0.82	0.34	0.02	0.003	0.06	0.04	34	0.005	8.2	0.003	17.1
SW-61	10/18/2012	TC	30.5	286	8.26	0.83	0.36	0.01	0.003	0.07	0.04	34	0.003	7.8	0.003	17.2
SW-62	10/18/2012	TC	30.0	329	8.3	0.79	0.35	0.01	0.007	0.06	0.04	34	0.004	7.5	0.003	17.0
SW-64	10/19/2012	TC	30.6	289	8.2	0.71	0.31	0.02	0.005	0.06	0.04	34	0.003	6.9	0.003	15.4
TC-09	10/23/2013	TC	26.8	200	7.01	0.85	0.40	0.02	0.003	0.07	0.03	46	0.013	3.8		19.7
TC-10	10/24/2013	TC	28.6	180	7.62	*2.31*	1.10	0.03	*0.028*	0.19	0.05	53	0.022	14.5	0.023	42.8
TC-11	10/25/2013	TC	28.2	121	6.46	1.05	0.48	0.02		0.08	0.03	48		5.7		23.6
SW-13	5/17/2012	TC-May	32.6	457	6.36	*31.69*	16.85	0.05	*0.025*	2.45	0.18	249	0.004	387.5	0.008	736.6
SW-23	5/20/2012	TC-May	32.5	461	7.31	*32.69*	17.46	0.05	*0.018*	2.57	0.18	255	0.001	398.7	0.008	763.8
SW-28	5/21/2012	TC-May	32.6	496	7.24	*33.58*	17.95	0.05	0.008	2.62	0.16	259	0.003	409.9	0.009	781.6
SW-32	5/22/2012	TC-May	32.3	415	7.37	*30.89*	16.48	0.05	*0.018*	2.63	0.17	260	0.007	412.2	0.008	782.8
SW-36	5/23/2012	TC-May	32.3	448	7.2	*33.62*	18.09	0.05	*0.016*	2.66	0.17	262	0.000	413.4	0.007	790.2
SW-56	5/7/2013	TC-May	30.3	179	7.4	*30.94*	17.18	0.07	0.002	2.84	0.18	232		299.3	0.025	685.3
TC-01	5/4/2013	TC-May	31.0	213	7.64	*30.23*	16.51	0.06	*0.034*	3.05	0.17	230	0.008	304.3	0.022	840.2
TC-02	5/5/2013	TC-May	28.9	217	7.85	*9.15*	4.71	0.05	*0.056*	2.60	0.30	198	0.007	245.0	0.021	663.1
TC-03	5/5/2013	TC-May	30.1	209	7.56	*32.27*	18.07	0.06	*0.022*	3.68	0.16	247		331.9	0.021	922.3
TC-04	5/5/2013	TC-May	30.9	124	7.55	*30.42*	16.66		0.003	3.18	0.20	318	0.080	298.1	0.662	837.2
TC-05	5/5/2013	TC-May	31.0	168	7.44	*31.45*	17.25	0.07	*0.030*	3.57	0.17	245	0.009	319.5	0.027	887.0
TC-06	5/10/2013	TC-May	31.6	183	7.47	*30.77*	16.64	0.07	*0.021*	3.03	0.16	240		317.3	0.030	886.3
TC-07	5/10/2013	TC-May	33.1	220	7.45	*24.87*	12.79	0.10	0.008	2.58	0.31	229	0.075	274.4	0.022	759.1
TC-08	5/4/2013	TC-May	31.6	115	8.29	*10.76*	5.31	0.05	*0.027*	0.88	0.29	162	0.014	92.3	0.013	233.3

Loc	Date	Mn	Mo	Na	P	S	Si	Sr	F	Cl	Br	NO_3_^−^	HCO_3_^−^	DIC	DOC	CIB (%)
Blank 1	10/19/2012			1		0.1				1		0.169	2.4	0.5	2.1	
Blank 2	10/18/2012			0.3						0.4					4.9	
Blank 3	5/13/2012			1		0.7			0.316	1			1.1	0.213	4.9	
Blank 4	5/13/2012	0.01		14	0.007		1.6	0.0085	1.97	10			0.4	0.07	4.5	
SW-06	5/14/2012	0.025	0.004	265	0.01	51.0	3.6	0.31	2.8	392	0.7				13.1	
SW-06	10/15/2012	0.011	0.001	190	0.01	37.8	3.3	0.22	0.1	283	1.5		101	20.0	3.2	4
SW-06	10/24/2013	0.002		154		18.1	2.3	0.16		233	0.5	1.2	115	22.7	8.8	4
SW-07	10/15/2012	0.035	0.002	892	0.19	55.8		0.56		1375			155	30.6	12.2	6
SW-08	5/16/2012	0.008	0.003	248	0.02	82.6	4.2	0.34	2.9	332					12.5	
SW-08	10/15/2012	0.001	0.002	195		66.2	3.6	0.27	0.1	296	1.4	0.2	123	24.2	10.6	1
SW-101	5/7/2013	0.144	0.006	2087	0.03	458.8	3.0	1.95	0.6	3297	12.7		190	37.4	24.7	1
SW-11	5/17/2012	0.141	0.005	163	0.16	30.3	4.2	0.27	2.2	220					13.0	
SW-12	5/17/2012	0.021	0.006	93	0.08	58.6	6.5	0.21	3.0	123					22.7	
SW-12	10/16/2012	0.050	0.002	290	0.02	93.5	5.5	0.31	0.1	377	1.6		127	25.0	5.7	10
SW-17	5/18/2012	0.168	0.006	380	0.10	67.0	5.5	0.30	2.6	506	1.4				12.6	
SW-17	10/16/2012	0.002	0.003	344	0.27	63.9	2.6	0.30		449			148	29.2	10.9	7
SW-17	5/7/2013	0.004		495	0.08	62.4	4.3	0.34	0.6	763	7.5		200	39.4	14.4	−1
SW-17	10/25/2013	0.013		286		43.7	3.1	0.21		419	0.6		166	32.7	11.5	0
SW-18	5/18/2012	0.073	0.002	369	0.04	45.6	5.4	0.34	3.5	551					14.4	
SW-18	10/16/2012	0.008	0.002	291	0.01	33.1	2.9	0.29	0.0	435	1.9		162	31.8	12.2	5
SW-18	5/7/2013	0.187	0.003	410	0.06	42.2	4.1	0.38	0.5	670	7.4		140	27.6	9.7	2
SW-18	10/25/2013			192		15.7	3.1	0.19		286	0.5		152	29.9	8.6	5
SW-19	5/19/2012	0.835	0.002	284	1.47	19.5	18.3	0.32	3.3	385					24.6	
SW-19	10/16/2012	0.120	0.001	251	0.92	14.6	16.7	0.24		371	1.7		226	44.5	8.9	7
SW-19	5/6/2013	0.358	0.003	429	0.40	37.1	6.8	0.43	0.4	693	7.4		220	43.3	20.0	6
SW-19	10/23/2013	0.190		319	0.52	15.7	12.9	0.30		493	0.6	0.7	180	35.4	10.1	1
SW-21	5/20/2012	0.044	0.005	230	0.06	57.6	4.5	0.28	2.3	305					10.1	
SW-21	10/17/2012	0.038	0.002	173	0.02	46.1	3.3	0.21	0.1	256	1.4	0.2	151	29.7	6.7	−1
SW-22	5/20/2012	0.112	0.003	229	0.01	57.3	4.2	0.28	1.8	302					12.9	
SW-22	10/26/2013	0.035		158		28.3	1.8	0.18		232	0.5	0.7	121	23.8	8.0	4
SW-25	5/20/2012	0.025	0.002	876	0.02	75.2	4.1	0.64	4.3	1279	3.8				12.5	
SW-25	10/17/2012	0.003	0.001	316	0.01	23.9	2.9	0.24	0.1	485	2.0		102	20.2	7.8	5
SW-25	5/7/2013	0.245		745	0.02	41.7	4.4	0.48	0.5	1152	8.4		143	28.2	12.9	4
SW-27	5/21/2012	0.002	0.003	552	0.03	34.3	2.3	0.34	3.8	810					9.3	
SW-27	10/19/2012	0.019	0.001	317	0.02	23.0	1.9	0.21	0.2	498	2.0		113	22.2	9.0	3
SW-27	5/5/2013	0.150	0.005	534	0.01	24.8	3.3	0.30	0.7	806	7.7	2.0	171	33.6	11.5	3
SW-27	10/26/2013	0.072		196		13.1	2.1	0.11		300	0.5		85	16.7	9.5	3
SW-30	5/22/2012	0.005	0.005	356	0.02	38.5	3.1	0.24	3.9	484					20.0	
SW-30	10/19/2012	0.017	0.003	219	0.01	44.0	3.2	0.22	0.1	330	1.6		143	28.1	6.4	1
SW-30	5/8/2013	0.017	0.009	387	0.02	62.2	2.3	0.35	0.6	578	7.2		206	40.6	15.0	0
SW-33	5/23/2012	0.452	0.006	2090	0.12	173.1	5.6	1.11	9.5	2840					34.2	
SW-33	10/18/2012	0.006	0.002	400	0.07	71.6		0.36		602			142	27.9	7.2	3
SW-33	5/5/2013	0.443	0.006	1287	0.11	139.5	4.2	1.00	0.7	2123	10.7		329	64.8	18.3	0
SW-33	10/27/2013	0.013		296		30.2	3.2	0.25		434	0.6	0.6	141	27.9	9.0	5
SW-52	10/15/2012	0.001	0.002	195	0.08	53.3	5.3	0.23	0.1	298	1.4		112	22.1	3.9	3
SW-52	10/24/2013	0.001	0.002	162	0.00	44.9	4.2	0.21		249	0.5		128	25.1	8.7	6
SW-63	10/19/2012	0.008	0.002	368	0.22	25.5	3.6	0.21	0.2	500	2.0	0.4	106	20.8	6.2	8
SW-70	10/27/2013	0.117		123		34.6	0.1	0.16		188	0.5	0.6	96	18.9	7.8	3
SW-09	10/15/2012	0.249	0.006	659		39.8		0.49		922			273	53.8	12.5	7
SW-14	10/25/2013	0.006		461		62.6		0.00		661	0.6	0.2	262	51.5	11.8	−14
SW-16	10/16/2012	0.005	0.001	84	0.01	11.5	3.9	0.19	0.1	119	1.0		118	23.2	4.6	6
SW-20	10/16/2012	0.138	0.001	259	0.02	21.9	2.5	0.37	0.1	400	1.8	0.2	104	20.4	5.6	11
SW-20	10/23/2013	0.063		111		6.1	3.2	0.21		168	0.5		219	43.0	8.0	2
SW-24	10/17/2012	0.024	0.001	229	0.02	14.6	2.6	0.24	0.1	353	1.7	0.2	126	24.8	5.1	6
SW-31	10/18/2012	0.009	0.002	325	0.01	33.9	2.5	0.32	0.1	504	2.1	0.2	125	24.7	7.1	4
SW-31	10/27/2013	0.029		267		20.2	2.8	0.20		395	0.6	0.6	97	19.1	7.3	5
SW-34	10/18/2012	0.022	0.001	387	0.01	26.9	0.3	0.36		523			154	30.3	8.0	11
SW-35	10/16/2012	0.045	0.001	208	0.01	13.6	1.5	0.20	0.1	313	1.6	0.3	98	19.4	4.7	7
SW-35	10/23/2013	0.136		217		8.0	2.5	0.33		324	0.6		266	52.4	9.9	3
SW-51	10/15/2012	0.003	0.001	255	0.01	66.2	0.7	0.30	0.1	358	1.6	0.2	126	24.7	5.7	2
SW-53	10/15/2012	0.015	0.001	110	0.03	11.9	4.2	0.16	0.1	163	1.1	0.3	109	21.5	3.8	6
SW-53	10/25/2013	0.093		215		18.2	2.6	0.25		332	0.6		176	34.7	9.3	4
SW-55	10/17/2012	0.260	0.006	299	0.04	55.2	0.5	0.35	0.1	432	1.8		111	21.9	4.7	7
SW-59	10/17/2012	0.005	0.002	700	0.04	144.0		0.67		905			109	21.4	7.0	10
SW-60	10/17/2012	0.006	0.002	711	0.03	142.4		0.66		1087			116	22.8	6.5	3
SW-65	10/19/2012	0.072	0.001	204	0.03	22.3	3.5	0.24	0.1	313	1.5		140	27.6	6.4	4
RW-02	10/26/2013			2		0.3				2		0.2			1.8	19
RW-03	10/26/2013			1		0.2				2			3	0.6	1.0	−13
RW-04	10/27/2013			3		0.9	2.9	0.02		4		0.1	35	6.9	1.7	−2
SW-07	5/16/2012	0.002	0.007	9917	0.02	729.3	1.2	4.36	27.6	13,678	14.6				19.2	
SW-09	5/16/2012	0.002	0.004	8642	0.03	565.1	0.4	3.27	23.3	12,105	13.8				20.6	
SW-10	5/16/2012	0.607	0.006	7559	0.15	557.5	4.5	3.61	22.5	10,686	21.1				23.6	
SW-100	5/4/2013	0.011	0.007	7471	0.07	603.7	2.7	4.75	0.8	11,415	37.9		188	37.0	6.9	5
SW-103	5/7/2013	0.006	0.005	1737	0.02	489.0	1.3	1.87	0.7	2587	10.2	1.5	101	19.8	15.8	1
SW-14	5/18/2012	0.775	0.009	6395	0.03	507.0	2.4	3.45	20.0	8718	15.7				45.9	
SW-16	5/18/2012	0.006	0.010	10,137	0.04	665.0	2.1	4.13	29.4	14,085	15.7				21.1	
SW-20	5/19/2012	0.044	0.008	9724	0.06	607.4	1.7	3.74	27.5	13,247	25.3				34.0	
SW-20	5/6/2013	0.040	0.006	6406	0.07	555.0	0.0	4.33	0.8	10,475	34.9		157	31.0	7.6	2
SW-24	5/20/2012	0.038	0.003	8565	0.02	522.1	2.2	3.47	25.0	11,926	21.1				19.2	
SW-24	5/7/2013	0.351	0.004	7419	0.07	588.9	3.1	4.69	0.8	11,537	38.2		203	40.0	8.0	4
SW-29	5/21/2012	0.044	0.006	6705	0.05	668.3	0.7	3.36	20.4	9027	20.2				43.1	
SW-29	5/6/2013	0.011		5439	0.22	77.5	1.3	3.69	0.4	8429	29.3		135	26.7	22.4	8
SW-31	5/22/2012	0.022	0.005	11,760	0.01	749.8	0.9	4.92	33.0	16,032	32.9				18.1	
SW-31	5/5/2013	0.229	0.004	8224		759.7	1.0	5.62	0.8	12,705	42.0	1.8	107	21.1	9.1	5
SW-34	5/23/2012	0.297	0.013	10,886	0.03	731.1	1.4	4.82	30.3	15,677	32.6				14.0	
SW-35	5/19/2012	0.734	0.009	9736	0.07	626.9	2.1	4.29	27.5	13,526	23.8				33.9	
SW-35	5/6/2013	0.010	0.004	7266	0.10	559.3	1.4	4.22	0.7	11,182	37.1		178	35.1	10.1	5
SW-105	10/22/2013	0.125		73		6.7	4.0	0.16		112	0.5		161	31.7	8.8	3
SW-50	10/15/2012	0.001	0.001	57	0.08	7.3	4.3	0.13	0.1	86	0.9	0.5	138	27.1	8.0	−1
SW-58	10/17/2012	0.002	0.001	123	0.05	12.7	4.0	0.17	0.1	182	1.2		107	21.2	7.9	6
SW-61	10/18/2012	0.001	0.001	120	0.04	11.8	4.3	0.17	0.1	179	1.2	0.5	113	22.3	3.1	5
SW-62	10/18/2012	0.001	0.001	112	0.05	11.4	4.4	0.17	0.1	166	1.1	0.6	114	22.5	4.5	6
SW-64	10/19/2012	0.000	0.001	108	0.06	10.4	4.3	0.16	0.1	143	1.1		115	22.7	4.9	8
TC-09	10/23/2013	0.146		95		3.9	4.0	0.19		143	0.5		226	44.6	9.0	1
TC-10	10/24/2013	0.008	0.001	373	0.06	41.2	1.2	0.34		543	0.6	0.2	183	36.0	8.5	4
TC-11	10/25/2013	0.008		127		18.6	3.0	0.21		195	0.5		174	34.3	8.6	2
SW-13	5/17/2012	0.002	0.009	9965	0.07	666.4	1.7	4.30	26.6	13,879	25.3				9.6	
SW-23	5/20/2012	0.002	0.009	10,347	0.05	684.4	1.7	4.49	28.6	14,309	15.5				8.2	
SW-28	5/21/2012	0.001	0.007	10,384	0.05	703.0	1.6	4.57	28.8	14,765	29.0				11.6	
SW-32	5/22/2012	0.005	0.012	10,358	0.04	705.1	1.7	4.57	30.8	14,904	27.0				10.9	
SW-36	5/23/2012	0.001	0.007	10,670	0.06	711.2	1.7	4.61	29.9	14,811	29.9				9.3	
SW-56	5/7/2013	0.002	0.008	7843	0.05	620.9	1.7	4.87	0.8	11,763	39.1		171	33.6	4.7	5
TC-01	5/4/2013	0.001	0.007	7400	0.04	653.7	1.5	4.98	0.9	11,909	39.7		150	29.5	5.6	4
TC-02	5/5/2013		0.006	5765	0.07	516.7	1.9	3.96	0.8	9219	31.0	1.5	160	31.5	5.4	4
TC-03	5/5/2013		0.005	7739	0.21	714.1	1.5	5.41	0.9	12,552	41.7		158	31.1	4.2	4
TC-04	5/5/2013	0.007	0.030	7492	0.36	674.6		5.66	0.8	11,899	39.6		171	33.6	4.6	5
TC-05	5/5/2013	0.001	0.006	7551		697.9	1.7	5.31	0.9	12,154	40.4		165	32.4	4.3	4
TC-06	5/10/2013	0.001	0.006	7423	0.06	693.1	1.5	5.19	0.9	12,120	40.3		159	31.3	4.7	3
TC-07	5/10/2013	0.211	0.006	6368	0.08	596.4	2.2	4.53	0.8	10,561	35.2		160	31.5	6.1	3
TC-08	5/4/2013	0.003	0.005	2473	0.11	251.0	2.8	1.77	0.7	3926	15.4		224	44.2	13.4	3

All concentrations in mg/L. Missing values correspond to concentrations below detection. Detection limits reported in [12]. DIC not measured for May 2012 samples, so charge imbalance error (CIB) not calculated

Salinity calculated from specific conductivity (SpC) using calculator at http://www.chemiasoft.com/chemd/salinity_calculator, after Standard Methods for the Examination of Water and Wastewater, 20th edition, 1999

Specific conductivity values that exceed the Bangladesh Government guideline of 2 mS/cm and arsenic concentrations that exceed the World Health Organization guideline of 0.01 mg/L are italicized

Water types: *FP* freshwater pond, *RP* rice paddy, *SP* shrimp pond, *TC-Oct.* tidal channel October, *RW* rainwater

Water samples were collected by rinsing a 1 L bottle, filling it, and immersing the Hydrolab Sonde for field measurements. Rainwater samples were collected in clean glass dishes set out just before a rain event. Next, a syringe with a 0.45 μm filter was used to withdraw 30 mL and transfer it to a polyethylene sample bottle for inductively coupled plasma (ICP) analysis. One drop of concentrated nitric acid (HNO_3_) was added to the bottle. Another 60 mL was filtered and placed in a sample bottle without acid for ion chromatography (IC) and total organic carbon (TOC) analysis (except for samples collected in May 2012).

Figure 1 lists the five types of surface water sampled and shows sample locations. In total, 44 freshwater pond samples, 18 shrimp pond samples, 18 rice paddy water samples, and 23 tidal channel samples were collected (Table 2). Indicative of seasonal land use, shrimp ponds were present only in the dry season and rice paddies only in the wet season. In October 2013 three rainwater samples were collected.

Continuous measurement of surface water salinity was collected from March 2012 until February 2013 by a Schlumberger Water Service Technologies CTD-Diver deployed in the Bhadra River, a tidal channel close to the Sundarbans and Polder #32 study areas (see Fig. 1 for location, 22°27′36.9″N 89°28′09.6″E). This Diver (model DI271) measures and records conductivity, temperature, and depth and has the following rated accuracy and precision: pressure measurement range up to 10 m, accuracy of 0.5 cm and resolution of 0.2 cm; conductivity measurement range of 0–120 mS/cm, with accuracy of ±1% of the reading with a minimum of 10 µS/cm, and resolution of 0.1% of reading with minimum of 1 µS/cm for 30 mS/cm range and 10 µS/cm for 120 mS/cm range; temperature measurement range −20 to 80 °C, accuracy of 0.1 °C and resolution of 0.01 °C. The Diver was attached to a ¾ inch (1.9 cm) diameter metal rebar mount, deployed and positioned ~15–20 cm above the bed of the tidal channel, below the spring tide low water line. Measurements were recorded every 10 min during the deployment period (N = 43,832). Measured conductivity and temperature was then used to calculate surface water salinity using standard water quality equations (e.g., [35]).

### Water chemistry

#### Water analysis

For all analyses an analytical blank and check standard was run every 10–20 samples and required to be within 15% of the specified value. If the maximum concentration in the calibration standards was exceeded, then samples were diluted gravimetrically to within the targeted analytical range.

Acidified aqueous samples were analyzed for metal cation concentrations using a Varian ICP Model 720-ES ICP-OES utilizing EPA Method 6010B. Five-point standard curves were used for an analytical range between approximately 0.1 and 25 mg/L for trace metals and approximately 0.1 and 500 mg/L for major ions.

Elements below detection were reanalyzed using a Perkin Elmer Elan 6100 DRC II ICP-MS in both standard and dynamic reaction chamber (DRC) modes. Standard analysis mode was used for all analytes except for As and Se, which were run in DRC mode with 0.5 mL/min of oxygen as the reaction gas. Seven-point standard curves were used for an analytical range between approximately 0.5 and 250 µg/L and completed before each analysis.

Analyses of anions were performed on unacidified samples using a Metrohm 881 Compact IC Pro employing ASTM Method D-4327-03. Seven-point calibration curves were generated by dilution of a multi-anion standard at 500×, 200×, 100×, 50×, 10×, 2×, and 1× and were accepted with a correlation coefficient of at least 0.995. A volume of approximately 10 mL of undiluted sample was loaded for analysis.

Analyses of organic and inorganic carbon were performed on unacidified samples using a Shimadzu model TOC-V CPH/CPN using ASTM Method D-7573-09. Five-point calibration curves, for both dissolved inorganic carbon (DIC) and non-purgeable DOC, were generated for an analytical range between 5 and 100 ppm and were accepted with a correlation coefficient of at least 0.995. A volume of approximately 20 mL of undiluted sample was loaded for analysis. DIC analysis was performed first for the analytical blank and standard and then the samples. DOC analysis was carried out separately after completion of DIC analysis. DOC analysis started with addition of 2 M hydrochloric acid to achieve a pH of 2 along with a sparge gas flow rate of 50 mL/min to purge inorganic carbon prior to analysis.

#### Quality assurance/quality control

Analysis of May 2012 nitrate NO_3_^−^ and DIC concentrations was compromised due to addition of HNO_3_ (i.e., unacidified samples were not collected in May 2012). Therefore, results for May 2012 NO_3_^−^ and HCO_3_^−^ concentrations are not used in the data analysis nor can charge-balance errors or saturation indices be determined for May 2012 samples.

To calculate charge balance errors PO_4_^3−^ concentrations were calculated from the P concentration measured by ICP and SO_4_^2−^ concentrations from S concentrations measured by ICP. Measured DIC values were used to calculate concentrations of HCO_3_^−^. For samples with complete chemical analyses (excludes May 2012 samples) the average charge-balance error was 3.9%.

Method detection limits are reported in Ayers et al. [12]. Sample blanks consisting of deionized water were collected in the field and analyzed, yielding method blank sample concentrations that were consistently below analytical detection limits.

### Data reduction

Mineral saturation indices were calculated for select samples using the Spec8 program in the Geochemists Workbench v. 9 and the default thermodynamic database thermo.dat [36]. Principal components analysis in SPSS was used to reduce the number of dimensions (variables) needed to describe the Polder 32 compositional data for surface water (this study) and groundwater samples [12]. Variables that were not normally distributed or had missing values were eliminated, as were samples that were compositional outliers (GW-42). For May (dry season) and October (wet season) data this left 190 samples and 13 variables. When measured concentrations were below detection in the two rainwater samples (RW-02 and RW-03) we substituted the method detection limit for the concentration. The output consisted of loadings (coefficients of the eigenvectors) for the two principal components factors PC1 and PC2. Loadings measure the extent to which a factor is associated with a variable [37]. The factor scores for each sample were calculated by normalizing the original variables to standard scores or z values:

1 $${\text{z }} = \left( {{\text{x }}{-} \upmu } \right)/\upsigma$$

where μ is the mean and σ the standard deviation for that variable. The z value vector was then multiplied by the appropriate coefficient vector.

## Results

Results of water analyses are presented in Table 2, and a summary of key water quality parameters is in Table 3. Time elapsed between sample collection and analysis on nonacidified samples ranged between 12 and 38 days, but no significant change in measured concentrations of DOC or DIC were observed over time, although nitrate decreased. Furthermore, measurements of DOC in acidified and nonacidified samples were not significantly different.

**Table 3:** Summary of key water quality parameters classified by water type

Parameter	Blank		Fresh water pond		Rice paddies		Rain water		Shrimp ponds		Tidal channel Oct.		Tidal channel May		Tube well	
Number n	4		44		18		3		18		9		14		81	
	Avg.	1 σ	Avg.	1 σ	Avg.	1 σ	Avg.	1 σ	Avg.	1 σ	Avg.	1 σ	Avg.	1 σ	Avg.	1 σ
Eh (mV)			273	126	276	77	173	68	325	162	246	78	279	141	69	97
pH			8.2	0.6	8.2	0.7	7.3	1.2	8.1	0.4	7.8	0.8	7.4	0.4	6.9	0.4
Salinity (ppt)			1.02	0.69	0.91	0.55	0.02	0.02	14.76	3.91	0.43	0.26	15.14	4.48	3.64	2.00
Geom. mean As (μg/L)	−2.22		−2.0	0.3	−2.3	0.4			−1.8	0.4	−2.4	0.4	−1.8	0.4	−1.4	0.6
Geom. mean S (mg/L)	−0.63	0.67	1.6	0.3	1.4	0.4	−0.4	0.3	2.7	0.2	1.0	0.3	2.8	0.1	0.7	0.8
Geom. mean DOC (mg/L)	0.59	0.18	1.0	0.2	0.8	0.1	0.2	0.1	1.2	0.3	0.8	0.2	0.8	0.2	1.4	0.2
>10 μg/L As			41%		22%		0%		78%		11%		71%		83%	
>50 μg/L As			2%		0%		0%		11%		0%		7%		47%	
>2 mS/cm			36%		28%		0%		100%		11%		100%		100%	

For each water type, measured concentrations of most elements displayed a lognormal distribution. This was confirmed by transforming the concentrations to their base 10 logarithms and testing for normality using Kolmogorov–Smirnov tests. All statistical tests and plots therefore use log_10_ values of concentrations. Parametric statistical tests were used unless their assumptions were violated (e.g., non-normal distributions), in which case equivalent nonparametric tests were used. Cutoffs for statistical tests are at a significance level P = 0.05, meaning that any differences referred to in the following discussion are significant at the 95% level. Uncertainties in normally distributed parameters are reported as one standard deviation (1σ).

### Tidal channels

**Fig. 4:**
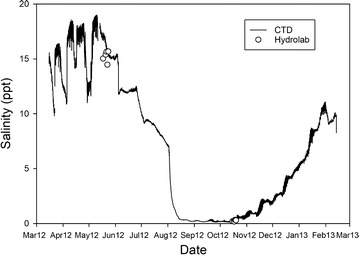
Continuous CTD measurements of tidal channel salinity over a 1-year period, from March 2012 to February 2013, show salinity variation throughout the wet and dry seasons. Discrete measurements made using the Hydrolab in May and October 2013 are shown by *open black circles*

The continuous measurements of tidal channel salinity from the CTD (location in Fig. 1) were compared with discrete measurements made using a Hydrolab in May and October 2013 (various locations shown in Fig. 1). The two different methods show good agreement (r^2^ = 0.99), with an average difference of 8% between measurements made at the same times likely caused by spatial variability (Fig. 4). Salinity measurements show that the tidal channels surrounding Polder 32 were brackish at the beginning of the deployment period (March to May), during the winter dry season in southwest Bangladesh. While there was much heterogeneity from March to May, the salinity in the tidal channels was on average 15 ppt, exhibiting slightly higher salinity, 17–19 ppt, during spring tides and slightly lower salinity, 10–13 ppt, during neap tides. During the wet monsoon season (late May to August), surface waters gradually freshened in this region, reaching salinities as low as 0.15 ppt and remaining low until October.

Because tidal channel samples have much higher salinities in May than in October, water samples were divided into “tidal channel May” and “tidal channel October” groups. Four samples collected in October 2013 from irrigation channels that connect rice paddies to the tidal channels (samples SW-105, TC-09, TC-10, and TC-11) were not significantly different from October tidal channel samples, consistent with observations that the irrigation channels were hydraulically connected to the tidal channels. These samples were therefore classified as tidal channel October samples in the analyses (Table 2).

### Freshwater ponds

Besides tidal channels, the only other surface water type for which we have multiple samples in both May and October is freshwater pond. Freshwater pond samples in May have significantly higher SpC, As, Na, S, and DOC than in October, but no significant differences are observed for pH or P (Table 2). However, even significant differences are small. For example, average salinity was 1.3 ppt in May and 0.8 ppt in October (Table 3). Furthermore, histograms and normality tests suggest that all freshwater pond samples can be treated as a single population, which we do for simplicity.

### Comparison of water types

Freshwater pond and rice paddy samples are all found to be Na–Cl water type and oversaturated in dolomite and calcite. All shrimp pond samples are Na–Cl type and oversaturated in dolomite and calcite. One tidal channel sample is Na–HCO_3_ type while all others are Na–Cl type. All tidal channel samples are saturated in goethite, calcite, and dolomite.

**Fig. 5:**
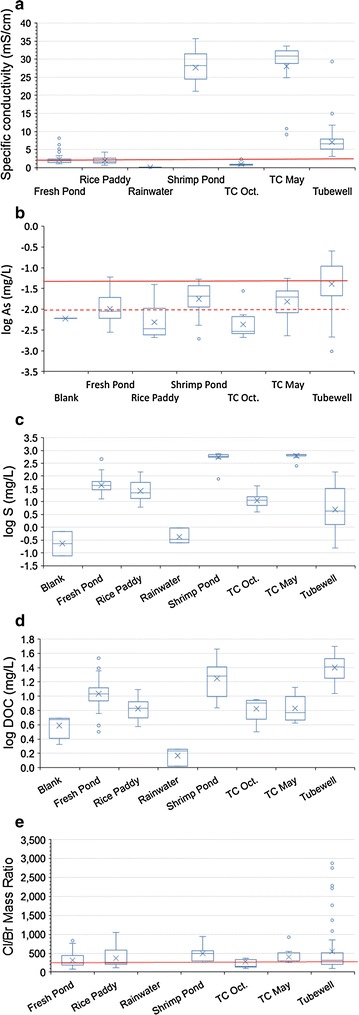
Box and whisker plots of water compositions classified by water type. “*TC*” indicates tidal channel. In all plots the *horizontal line* inside the box represents the median. The boxes’ lower boundary is the 25th percentile and upper boundary the 75th percentile. The sample mean is an “*x*” symbol. The “whiskers” extend to 1.5 times the interquartile range *above* and *below* the box, and outliers that plot outside the interquartile range are shown as *circles*. Tubewell groundwater sample data from [12]. **a** Specific conductivity in mS/cm measured in the field using a Hydrolab. For reference, the conductivity of pure seawater is ~50 mS/cm. The Bangladesh government drinking water guideline of 2 mS/cm is shown as a *horizontal red line*. **b** log_10_ values of arsenic concentrations in mg/L. The *solid red line* corresponds to the Bangladesh government drinking water guideline of 50 μg/L, and the *dashed red line* indicates the WHO guideline of 10 μg/L. Arsenic in rainwater was below detection. **c** log_10_ values of sulfur concentrations in mg/L. **d** log_10_ values of dissolved organic carbon concentrations in mg/L. **e** Cl/Br mass ratio. The *solid red lin*e corresponds to seawater

Using SpC as a measure of salinity, we observe three general salinity groupings for surface water (Fig. 5a). Shrimp pond and May tidal channel samples have very high and similar conductivities ~1/2 to 2/3 that of pure seawater (~30 mS/cm, pure seawater is 50 mS/cm = 35 ppt). Tubewell samples have intermediate conductivities (~5–10 mS/cm; all tubewell groundwater compositional data from [12]. All other surface water types have low conductivities (~1–3 mS/cm). In general, surface waters on and around Polder 32 have lower salinity in the wet season than in the dry season.

**Fig. 6:**
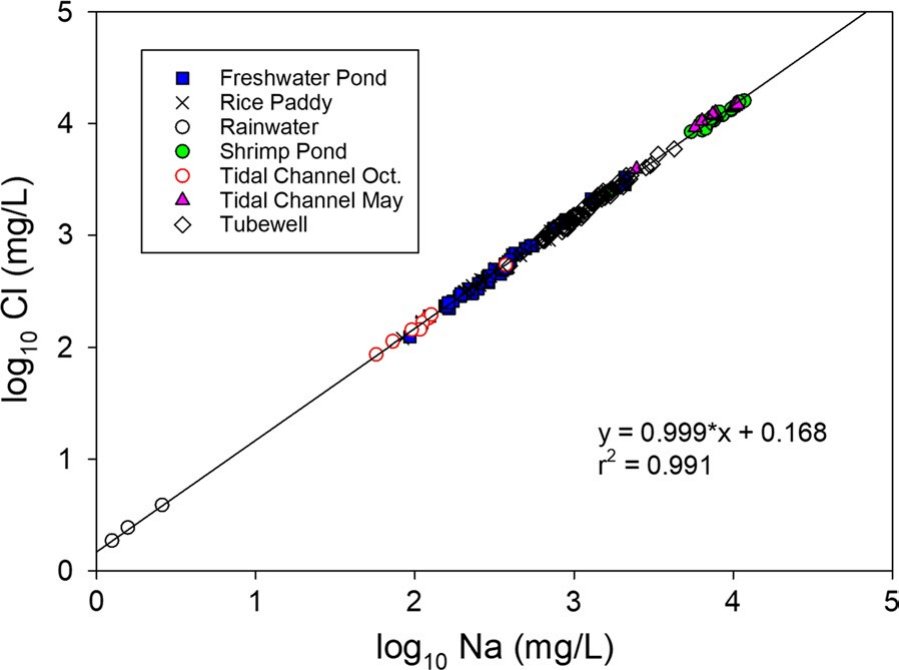
Scatter plot illustrating the correlation between log_10_ concentrations of conservative elements Na and Cl. Samples classified by water type

Concentrations of Na and Cl are positively correlated, and all water types plot on the same linear trend, indicating that these elements behave conservatively (Fig. 6). Dry season tidal channel and shrimp pond water samples have the highest concentrations and rainwater has the lowest concentrations of Na and Cl. Although they do not show perfect correlations, concentrations of B, K, S, Mg and Sr are well correlated with concentrations of Na and Cl, suggesting they also can be treated as conservative elements (Table 4, Additional file 1: Fig. S1). While S does not behave conservatively in TW samples [12], it does in all surface water samples because it occurs as sulfate ion under oxidizing conditions.

**Table 4:** Pearson correlation coefficient matrix for all surface water and groundwater samples

	Eh	H^+^	As	B	Ca	Fe	K	Mg	Na	S	Sr	Cl	HCO_3_^−^	DOC
Eh	1													
H^+^	−0.41	1												
As	−0.39	0.34	1											
B	0.09	−0.06	−0.04	1										
Ca	−0.13	0.34	0.15	0.53	1									
Fe	−0.38	0.27	0.18	−0.13	0.35	1								
K	0.38	−0.12	−0.13	0.91	0.55	−0.17	1							
Mg	0.22	−0.02	−0.08	0.94	0.70	−0.04	0.96	1						
Na	0.31	−0.05	−0.09	0.91	0.64	−0.11	0.97	0.96	1					
S	0.39	−0.23	−0.19	0.89	0.52	−0.22	0.96	0.92	0.92	1				
Sr	0.07	0.05	0.01	0.91	0.77	−0.02	0.88	0.96	0.92	0.85	1			
Cl	0.28	−0.05	−0.09	0.93	0.64	−0.11	0.97	0.98	1.00	0.92	0.94	1		
HCO_3_^−^	−0.69	0.38	0.59	0.03	0.22	0.35	−0.18	−0.09	−0.09	−0.29	0.02	−0.10	1	
DOC	−0.28	0.15	0.52	0.01	0.26	0.29	−0.05	−0.01	0.05	−0.11	0.03	0.02	0.87	1

In contrast, redox-sensitive species such as As, Fe, and DOC do not behave conservatively (Additional file 2: Fig. S2). Tubewell groundwater As concentrations are significantly higher and show greater variability than all other water types (Fig. 5b; Table 3). Dry season tidal channel water and shrimp pond water have intermediate As concentrations, followed by freshwater ponds, and then rice paddies and wet season tidal channel samples with the lowest As concentrations.

Sulfur shows very large differences in concentrations between water types (Fig. 5c). Sulfur concentrations in shrimp pond and dry season tidal channel water samples have very similar and by far the highest concentrations of all water types. Besides rainwater and blanks, groundwater samples from tubewells have the lowest sulfur concentrations.

The geometric mean DOC concentration in both May and October tidal channels of ~6.7 mg/L (Table 3) is almost identical to the world average for rivers of 5.8 mg/L [38], while the 18 mg/L in shrimp ponds and 11 mg/L in freshwater ponds are similar to the 12 mg/L world median in eutrophic lakes [39] (Fig. 5d). Groundwater samples have the highest geometric mean DOC concentration of 25 mg/L (Fig. 5d), much higher than the global groundwater median of 0.7 mg/L [39]. No correlations are observed between Eh, concentrations of reducing agents (DOC), and concentrations of elements with variable oxidation states (As, Fe, Mn, M, and S), indicating that redox disequilibrium is the norm.

### Principal components analysis

**Fig. 7:**
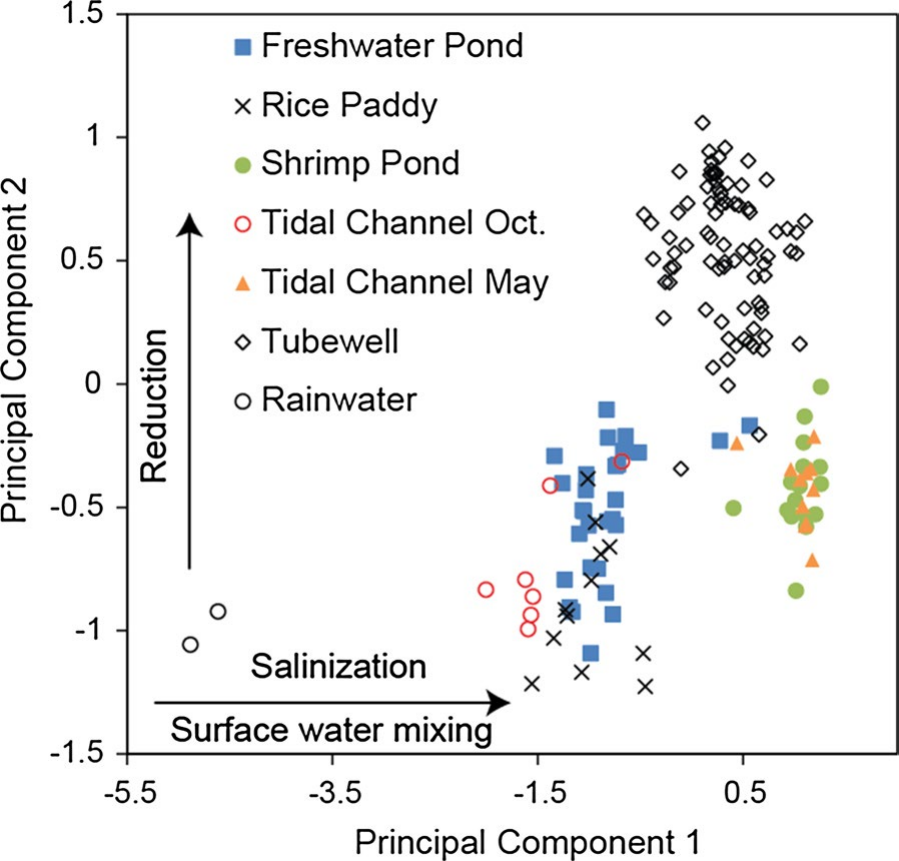
Plot of principal component scores of water samples classified by water type

A principal components analysis showed that only two factors PC1 and PC2 are needed to explain 80% of the compositional variance. For PC1 the resulting equation is:

2 $$\begin{aligned} {\text{PC1 }} &= - 0.012 {\text{z}}_{\text{Eh}} {-} 0.061 {\text{z}}_{\text{pH}} + 0.055 {\text{z}}_{\text{As}} + 0.132 {\text{z}}_{\text{B}} \\ & \quad + 0.13 {\text{z}}_{\text{Ca}} + 0.038 {\text{z}}_{\text{Fe}} + 0.13 {\text{z}}_{\text{K}} + 0.144 {\text{z}}_{\text{Mg}} \\ & \quad + 0.144 {\text{z}}_{\text{Na}} + 0.048 {\text{z}}_{\text{S}} + 0.144 {\text{z}}_{\text{Sr}} + 0.144 {\text{z}}_{\text{Cl}} \\ & \quad + 0.048 {\text{z}}_{\text{DOC}} \end{aligned}$$

where “z” is the z-score for each compositional variable. PC2 is calculated in an analogous fashion using the coefficients for factor 2 in Table 5. PC1 is plotted versus PC2 in Fig. 7.

**Table 5:** Principal components loadings

Factor	1	2
Eh (mV)	−0.012	−0.212
pH	−0.061	−0.187
log As (μg/L)	0.055	0.179
log B (μg/L)	0.132	−0.005
log Ca (μg/L)	0.13	0.011
log Fe (μg/L)	0.038	0.239
log K (μg/L)	0.13	−0.105
log Mg (μg/L)	0.144	−0.044
log Na (μg/L)	0.144	−0.033
log S (μg/L)	0.048	−0.228
log Sr (μg/L)	0.144	−0.02
log Cl (μg/L)	0.144	−0.031
log DOC (μg/L)	0.048	0.193

## Discussion

### Salinization

#### Tidal channels and shrimp ponds

Tidal channel water shows the greatest seasonal variation in composition of all water types, having much lower salinity during the monsoon due to dilution from freshwater sources (Figs. 4, 5a, 6). These variations in tidal channel salinity are due to variable degrees of mixing of Bay of Bengal seawater with the freshwater plume of the Ganges–Brahmaputra River, which mix on the shelf and are advected inland with the tides [40]. Wet-season runoff and discharge from the Gorai River also contribute to seasonal variability in tidal channel salinity. In contrast, compositions of dry season tidal channel and shrimp pond water are nearly indistinguishable (Fig. 5), even for nutrients such as phosphorus that are added to shrimp ponds as fertilizer (Table 2). For example, their very high and similar sulfur concentrations suggest that shrimp pond water is sourced from dry season tidal channels rather than tubewells, and that seawater sulfate is present under oxidizing conditions. Only DOC (Fig. 5d) and Mn show significantly different concentrations between dry season tidal channel and shrimp pond samples, and those differences can be attributed to nonconservative behavior (redox cycling or sorption).

The Bangladesh government guideline for the salinity of drinking water of 2 mS/cm [1] is exceeded by 11% of wet season tidal channel samples and 100% of shrimp pond and dry season tidal channel samples (Table 3). Shrimp ponds are present only in the dry season, and are always close to tidal channels. Their similar compositions and spatial and temporal proximities confirm that dry season tidal channels provide the water and dissolved salts in brine shrimp ponds (Fig. 5). Observations of sluice gates in embankments separating shrimp ponds from tidal channels and discussions with shrimp farmers confirmed this inference. At low tide shrimp ponds can be drained into the tidal channel, and at high tide tidal channel water can be added to the shrimp ponds. Since the only surface water types that are highly saline are May tidal channel and shrimp pond, and May tidal channel water is the source of salts in shrimp ponds, it is the most likely source of salts in all surface water types except rainwater (exhibited by the mixing curve in Fig. 6).

Just as they are not the ultimate source of salts in surface waters, shrimp ponds are unlikely to be the source of salts in groundwater. Much evidence supports this notion, including lack of a correlation between groundwater salinity and distance to nearest shrimp pond [12]. Like the rest of the polder, shrimp ponds are underlain by impermeable muds that cap the surface stratigraphy in this region, limiting or preventing surface recharge [12]. Furthermore, old ^14^C ages and low tritium contents in groundwater from the shallow aquifer beneath Polder 32 confirm that there is limited surface recharge that would allow shrimp ponds to contaminate the shallow aquifer [13]. Thus, the source of salts in the groundwater is connate tidal channel water from deposition of aquifer sands during the mid-late Holocene [12].

Surface muds also prevent movement of saline shrimp pond water into adjacent freshwater ponds. For example, on polder 31 in May 2012 the specific conductivity of freshwater pond SW-08 was 1.4 mS/cm, while 10 m away the specific conductivity of brine shrimp pond SW-09 was 28 mS/cm (Fig. 1). This observation that freshwater could be maintained in a pond adjacent to a saline shrimp pond suggests that surface deposits are impermeable enough (i.e., have a low enough hydraulic conductivity) to prevent the transfer of salts through meter-scale pond embankments.

#### Rice paddies

Rice paddies are inundated with surface water only in the wet season. Field observations and analysis of satellite imagery show that rice paddy water comes from irrigation channels sourced from inland streams connected to tidal channels (Figs. 1, 2, 3). DOC contents of water from rice paddies and tidal channels support this inference, as they are similar to each other but different from all other water types present in the wet season (Fig. 5d). However, the median specific conductivity is significantly higher for rice paddy water than for wet season tidal channel water (Fig. 5a). This can be explained by three different scenarios, as explained below.

One explanation for the salinity of rice paddy water being higher than in the wet season tidal channel water is that it is sourced from salts deposited in the soil during the 2 years of tidal inundation following the embankment failures during Cyclone Aila in 2009 [20]. These salts would have slowly leached into soil porewater, and then diffused into the overlying paddy water during subsequent rice cultivation seasons. If this happened, we would expect the salinity of rice paddy water to be higher in areas that were inundated than in those that were not, but there is no statistically significant difference in specific conductivity (sites that were inundated are listed in Table 1).

Similarly, the higher conductivity of rice paddy water may result from the land being used for brine shrimp aquaculture in the dry season. A previous 15-year study showed that alternating shrimp farming with rice farming caused increases in soil salinity and decreases in rice yield [11]. However, our data show no significant difference in rice paddy water conductivity between samples from rice paddies that were shrimp ponds (Table 2, locations classified as rice paddy in October and shrimp pond in May) and samples from rice paddies that were not. Rice paddy water therefore shows no strong evidence of salinization caused by shrimp farming. Salts in shrimp ponds are mostly removed by discharging shrimp pond effluents to tidal channels, preventing salinization of agricultural fields. Any remaining salts in the soil are likely flushed out during wet season irrigation of rice paddies, preventing salt accumulation. Despite these findings, the potential for soil salinization caused by shrimp farming cannot be excluded for all areas due to variation in local water sources and farming methods.

Finally, the higher conductivity of rice paddy water may result from evaporation. The geometric mean concentrations of conservative elements are uniformly ~3× higher in rice paddy water than in wet season tidal channel water (Fig. 6), suggesting they are concentrated about 3× by evaporation. Rice paddies are prone to evaporative concentration because they are relatively shallow and subject to more significant temperature variations, compared to their deeper irrigation channel and tidal channel counterparts (~15–20 cm versus ~100–500 cm, respectively). Given these lines of evidence, we conclude that evaporation is the main cause of elevated salinity of rice paddy water relative to its wet season tidal channel source.

#### Freshwater ponds

Freshwater ponds are filled by rainwater, but may become saline when salts are added during inundation and concentrated by evaporation. Because freshwater ponds are closed depressions with no outlets, the only way that salts can be removed is through water abstraction, which combined with dilution from precipitation would cause the water to gradually become less saline after an inundation event. Freshwater ponds that were in areas inundated following Cyclone Aila in 2009 were not significantly more saline than those that were not in inundated areas (Tables 1, 2). This was true in both wet and dry seasons, even in May 2012, only 2 years after inundation ended. Thus, abstraction and dilution erased any evidence of increased salinity in freshwater ponds caused by inundation.

The average molar Na/Cl in freshwater ponds is 1.05, close enough to one to suggest that Na and Cl are added as NaCl, most likely in sea spray that returns to the surface during precipitation [41]. The concentrations of Na^+^ and Cl^−^ in freshwater ponds are 238× and 235× higher respectively than in rainwater, which makes evaporative concentration alone an unlikely explanation for the salinity of freshwater ponds being so much higher than in rainwater. However, because all water types have the same proportions of Na and Cl (Fig. 6), it is difficult to uniquely identify the salinity source in freshwater ponds. The Cl/Br mass ratio has been used to distinguish seawater (=290) from other salt sources with higher Cl/Br such as urine and West Bengal halite [42], but median and average Cl/Br for freshwater ponds are close to seawater (Fig. 5e). Seawater, whether in sea spray or in tidal channel water, is likely the ultimate source of salts in freshwater ponds, although molar Na/Cl in seawater is only 0.86 [43].

### Arsenic contamination

Arsenic concentrations are generally higher in groundwater than all surface water types (Fig. 5b), consistent with results from previous work [44]. Of surface water types, concentrations of As are highest in shrimp pond water and dry season tidal channel water it is derived from, but significantly lower in wet season tidal channel water (Table 3). In both dry and wet seasons As concentrations in tidal channel water are higher than the global average river water As concentration of 0.83 μg/L [45]. It is possible that As in tidal channel water is derived from groundwater added to the tidal channel, perhaps by groundwater irrigation of rice paddies upstream, since As concentrations are highest in groundwater. The higher concentration of As in tidal channel water in the dry season may result from a greater proportion of groundwater than in tidal channel water in the wet season when surface runoff and river discharge are high. Higher head gradients in the dry season may also cause greater discharge of reducing As-rich groundwater into tidal channels as baseflow [32].

Arsenic concentration exceeds 10 μg/L in 43% of freshwater ponds (Table 3). Arsenic may have been leached out of sediments lining the pond, especially if the pond was recently excavated, or it may have been added by tidal channel water during inundation. Deposition of As by groundwater seepage seems unlikely unless the mud cap was breached during pond excavation.

Of the 18 rice paddy water samples collected, 22% exceeded 10 μg/L, and the geometric mean As concentration was 5 μg/L (Table 3). Because rice paddies in Polder 32 are irrigated with tidal channel water and not groundwater, tidal channels are the local source of As. However, groundwater is likely the ultimate source of As, as it has the highest As concentrations. Much of the As added to rice paddies by groundwater or tidal channel water irrigation during the dry season is removed by floodwaters in the wet season [46]. However, poldering has reduced the frequency of inundation with fresh tidal channel water during the wet season, which may be causing As from irrigation water to accumulate in rice paddy soil and water. Still, rice paddy water As concentrations in Polder 32 are lower than in areas where groundwater from the shallow aquifer is used to irrigate rice paddies [47, 48].

### Compositional relationships between water types

Figure 6 shows that, for conservative elements, all water types can be formed by mixing of high salinity dry season tidal channel water with rainwater. Salinization of surface water is caused by tidal channel water inundation or irrigation, while the intermediate salt content of shallow groundwater is inherited from tidal channel water trapped in sediments during deposition [12]. Any deviation in composition from a dry season tidal channel water–rainwater mixture is due to nonconservative behavior, which primarily affects ions with multiple valence states. Nonconservative behavior is most apparent in groundwater compositions, which have lower Eh values than surface waters, and have much lower S concentrations than expected based on the proportions of tidal channel water and rainwater estimated from conservative element concentrations [12].

These observations suggest that only two factors explain most of the observed variation in compositions of all water types: salinization by mixing of saline tidal channel water with freshwater, followed by post-depositional progressive reduction of groundwater. Plotting principal components scores PC1 and PC2 for all samples classified by water type shows that PC1 increases with increasing dissolved salt content and therefore represents the process of salinization (Fig. 7). The conservative elements B, Ca, K, Mg, Na, Sr and Cl have the highest loadings on PC1 and are all positively correlated. PC2 represents progressive reduction, which affects the concentrations of nonconservative elements in groundwater. The PC2 scores for groundwater samples are higher than for surface water samples because groundwaters are more reducing (Fig. 7).

The principal components plot concisely summarizes the compositional relationships between the different water types. Since the PC factor scores are a measure of water composition, water types with similar compositions plot in the same areas. For example, dry season tidal channel water is compositionally similar to shrimp pond samples (Fig. 7), indicating that shrimp farmers draw water from the tidal channels for their shrimp ponds. Since the factor scores are calculated as linear combinations of compositional variables, mixtures plot on linear mixing lines connecting endmembers. Waters in wet season tidal channels, freshwater ponds and rice paddies can form as mixtures of dry season tidal channel water and rainwater. Groundwater compositions are distinct from all surface water types and could have formed by mixing of dry season tidal channel water and rainwater followed by reduction. The anomalously high DOC in groundwater is likely preserved from the connate water, as soil porewater in the Sundarbans has similarly high DOC [12, 49]. The DOC could also derive from surface sources such as freshwater ponds and shrimp ponds [50–52], which have high measured DOC concentrations (Fig. 5d).

The relationships between the concentrations of redox-sensitive species can be explained by examining their associated redox reactions. For iron:

3 $${\text{FeO}}\left( {\text{OH}} \right) + {\text{ 2 H}}^{ + } + = { 1}. 5 {\text{ H}}_{ 2} {\text{O }} + 0. 2 5 {\text{ O}}_{ 2} \left( {\text{aq}} \right) + {\text{ Fe}}^{ 2+ }$$

For sulfur:

4 $$2 {\text{ H}}^{ + } + {\text{ SO}}_{4}^{ 2- } = {\text{ H}}_{ 2} {\text{S}}\left( {\text{aq}} \right) + {\text{ 2 O}}_{ 2} \left( {\text{aq}} \right)$$

For carbon, where reduced organic matter is indicated by methane CH_4_:

5 $$8 {\text{ CH}}_{ 4} \left( {\text{aq}} \right) + {\text{ 16 O}}_{ 2} \left( {\text{aq}} \right) = {\text{ 8 HCO}}_{3}^{ - } + {\text{ 8 H}}_{ 2} {\text{O }} + {\text{ 8 H}}^{ + }$$

For arsenic, where the dominant forms of reduced (As(OH)_3_aq) and oxidized (HAsO_4_^2−^) arsenic correspond to the observed pH values of most surface waters:

6 $$2 {\text{ H}}^{+} +{\text{ HAsO}}_{4}^{ 2- } = {\text{ As}}\left( {\text{OH}} \right)_{ 3} \left( {\text{aq}} \right) + 0. 5 {\text{ O}}_{ 2} \left( {\text{aq}} \right)$$

Combining Eqs. (3)–(6) and indicating HAsO_4_^2−^ as being sorbed to goethite FeO(OH) so that it is immobile:

7 $$\begin{aligned} {\text{FeO}}\left( {\text{OH}} \right) & > {\text{HAsO}}_{4}^{ 2- } + {\text{ SO}}_{4}^{ 2- } + {\text{ 8 CH}}_{ 4} \left( {\text{aq}} \right) + { 13}. 2 5 {\text{ O}}_{ 2} \left( {\text{aq}} \right) \\ & = {\text{ H}}_{ 2} {\text{S }} + {\text{ Fe}}^{ 2+ } + 9.5{\text{ H}}_{ 2} {\text{O }} + {\text{ 2H}}^{ + } \\ & \quad + {\text{ 8 HCO}}_{3}^{ - } + {\text{ As}}\left( {\text{OH}} \right)_{ 3} \left( {\text{aq}} \right)\end{aligned}$$

Our geochemical interpretation is that reaction of DOC (represented simply as CH_4_) drives the reaction to the right, causing progressive reduction, reductive dissolution of ferric oxyhydroxide FeO(OH), and release of sorbed As, resulting in increased groundwater concentrations of dissolved As and Fe. At low Eh values H_2_S escapes or sulfides precipitate, decreasing aqueous S concentrations [12].

A correlation analysis for all surface water and groundwater samples is consistent with the compositional trends predicted by Eq. (7). In Table 4 in the row labeled “DOC” the signs of the Pearson correlation coefficient r values correspond to the signs of the stoichiometric coefficients in Eq. (7). As DOC is consumed as a reactant during progressive reduction, Eh and S decrease (negative coefficients), and Fe, As, H^+^, and HCO_3_^−^ increase (positive coefficients). Also, pairs of conservative elements have correlation coefficient values close to 1 (Table 4).

## Conclusions

In the area of Polder 32 in southwest Bangladesh drinking water sources include groundwater from the shallow aquifer and surface freshwater ponds. Groundwater is moderately saline (median salinity of 3.6 ppt). Freshwater ponds have lower salinity (1.1 ppt). All sampled surface waters are mixtures of tidal channel water and rainwater.

In the wet season rice paddy water is obtained from tidal channels via sluice gates along former stream channels and irrigation channels, but the low level of salts becomes concentrated ~3× by evaporation. In the dry season shrimp are farmed using saline tidal channel water. Alternating rice and shrimp farming in this area appears to have a negligible effect on rice paddy water composition and presumably on rice yields. Thus, in the area studied brine shrimp aquaculture can be sustainable if effectively managed. However, the WHO guideline of 10 μg/L As is exceeded by 83% of groundwater, 78% of shrimp pond, 71% of May tidal channel, 41% of freshwater pond, 22% of rice paddy, and 11% of October tidal channel samples. The high percentage of water samples that exceed the WHO guideline raises concerns about the arsenic content of shrimp grown in shrimp ponds, rice grown in rice paddies, and drinking water obtained from tubewells and freshwater ponds.

## Additional files

**Additional file 1: Figure S1.** Stacked histograms and bivariate scatter plots of concentrations of conservative elements.

**Additional file 2: Figure S2.** Stacked histograms and bivariate scatter plots of concentrations of non-conservative elements and the water quality parameter Eh, the oxidation–reduction potential.
