# Supplementary figures and images for: Salinization and arsenic contamination of surface water in southwest Bangladesh
Source: Geochem Trans. 2017 Sep 11;18:4. doi: 10.1186/s12932-017-0042-3 (PMC5593806; doi:10.1186/s12932-017-0042-3)

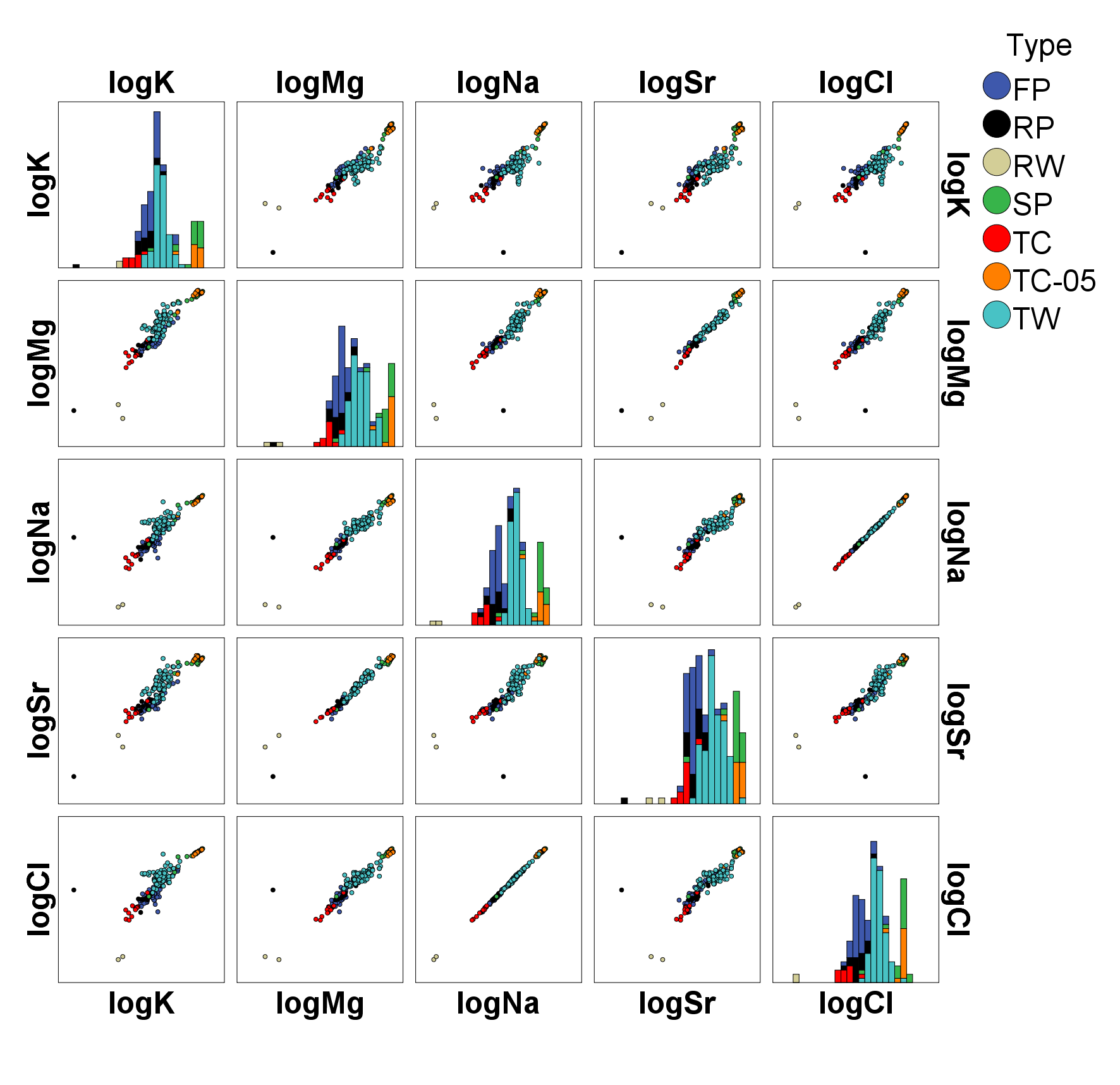

Supplement: Supplementary file 1 — Additional file 1: Figure S1. Stacked histograms and bivariate scatter plots of concentrations of conservative elements. [file 12932_2017_42_MOESM1_ESM.png]

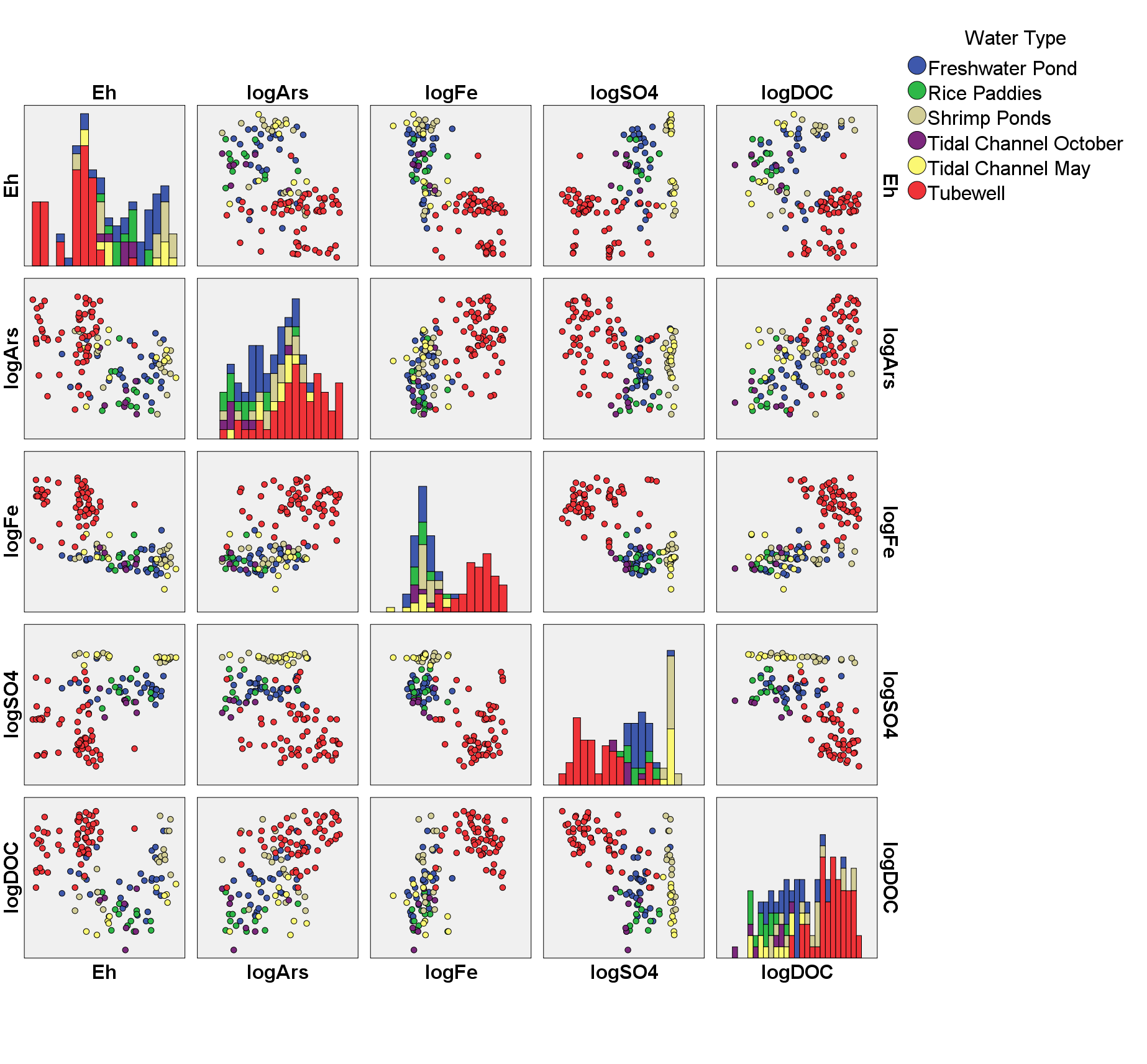

Supplement: Supplementary file 2 — Additional file 2: Figure S2. Stacked histograms and bivariate scatter plots of concentrations of non-conservative elements and the water quality parameter Eh, the oxidation–reduction potential. [file 12932_2017_42_MOESM2_ESM.png]
